# *In-silico* evaluation of natural alkaloids against the main protease and spike glycoprotein as potential therapeutic agents for SARS-CoV-2

**DOI:** 10.1371/journal.pone.0294769

**Published:** 2024-01-04

**Authors:** Mohibullah Shah, Ramsha Yamin, Iqra Ahmad, Gang Wu, Zainab Jahangir, Amen Shamim, Haq Nawaz, Umar Nishan, Riaz Ullah, Essam A. Ali, Sheheryar, Ke Chen

**Affiliations:** 1 Department of Biochemistry, Bahauddin Zakariya University, Multan, Pakistan; 2 Department of Infectious Diseases, The Affiliated Hospital of Southwest Medical University, Luzhou, China; 3 Department of Computer Science, University of Agriculture Faisalabad, Punjab, Pakistan; 4 Department of Chemistry, Kohat University of Science & Technology, Kohat, Pakistan; 5 Department of Pharmacognosy, College of Pharmacy, King Saud University, Riyadh, Saudi Arabia; 6 Department of Pharmaceutical Chemistry, College of Pharmacy, King Saud University, Riyadh, Saudi Arabia; 7 Department of Biochemistry and Molecular Biology, Federal University of Ceara, Fortaleza, Brazil; Ahram Canadian University, EGYPT

## Abstract

Severe Acute Respiratory Syndrome Corona Virus (SARS-CoV-2) is the causative agent of COVID-19 pandemic, which has resulted in global fatalities since late December 2019. Alkaloids play a significant role in drug design for various antiviral diseases, which makes them viable candidates for treating COVID-19. To identify potential antiviral agents, 102 known alkaloids were subjected to docking studies against the two key targets of SARS-CoV-2, namely the spike glycoprotein and main protease. The spike glycoprotein is vital for mediating viral entry into host cells, and main protease plays a crucial role in viral replication; therefore, they serve as compelling targets for therapeutic intervention in combating the disease. From the selection of alkaloids, the top 6 dual inhibitory compounds, namely liensinine, neferine, isoliensinine, fangchinoline, emetine, and acrimarine F, emerged as lead compounds with favorable docked scores. Interestingly, most of them shared the bisbenzylisoquinoline alkaloid framework and belong to *Nelumbo nucifera*, commonly known as the lotus plant. Docking analysis was conducted by considering the key active site residues of the selected proteins. The stability of the top three ligands with the receptor proteins was further validated through dynamic simulation analysis. The leads underwent ADMET profiling, bioactivity score analysis, and evaluation of drug-likeness and physicochemical properties. Neferine demonstrated a particularly strong affinity for binding, with a docking score of -7.5025 kcal/mol for main protease and -10.0245 kcal/mol for spike glycoprotein, and therefore a strong interaction with both target proteins. Of the lead alkaloids, emetine and fangchinoline demonstrated the lowest toxicity and high LD50 values. These top alkaloids, may support the body’s defense and reduce the symptoms by their numerous biological potentials, even though some properties naturally point to their direct antiviral nature. These findings demonstrate the promising anti-COVID-19 properties of the six selected alkaloids, making them potential candidates for drug design. This study will be beneficial in effective drug discovery and design against COVID-19 with negligible side effects.

## Introduction

The coronavirus disease of 2019 (COVID-19), a widespread and terrible virus, originated in Wuhan, China, brought on by the SARS-CoV-2 (Severe Acute Respiratory Syndrome Corona Virus 2), broke out in late December 2019, due to which thousands of people died worldwide. SARS-CoV-2 is named so because of its structural resemblance to numerous coronaviruses linked to acquired respiratory syndrome [1]. The following symptoms are frequently recorded in these patients: cold, fatigue, chest pain, fever, flu, and cough [2]. In addition to these typical symptoms, several patients claimed to have digestive issues also [3]. One of the major factors contributing to the death of COVID-19’s patients is respiratory failure. The adherence of coronaviruses to the epithelial tissues of the lungs results in the development of an immunological response and a cytokine storm, which in turn causes respiratory failure.

There are currently no approved medications for the treatment of COVID-19. Research is now being conducted to identify and create medications that can be used successfully against this lethal virus. SARS-COV-2 can carry out physiological functions using the synthetic machinery of the host cell, and targeting any stage of the viral life cycle can serve as an effective therapeutic target for the development of antiviral drugs [4].

The high death rate and absence of effective treatments for COVID-19 require the quick development of innovative medications, and computer-aided drug design is thought to be a key tool in the search for new therapeutics. Natural compounds [5], synthetic compounds [6], and commercially available drugs [7] have been studied through virtual screening and pharmacokinetic predictions for the treatment of COVID-19.

Hydroxychloroquine, remdesivir, lopinavir, chloroquine, favipiravir, ribavirin, and umifenovir, are a few antiviral medications that are used to treat COVID-19 effectively [8–10]. Similarly, the COVID-19 treatment guidelines panel advises using molnupiravir unless absolutely necessary. According to studies, molnupiravir medication significantly lowers hospitalization and mortality rates, particularly in the patients with the omicron subvariants of the virus [11]. Its function in broader COVID-19 therapeutic approaches and post-acute COVID-19 sequelae is still being under [12]. Approximately 200 FDA-approved medications are being used to treat COVID-19, including several antibacterial, anti-inflammatory, and antiviral medications. None of these medications have been recognized as effective treatments for COVID-19 due to their toxicity. All of these medications don’t have the potential to lesson or stop the development of this terrible disease [2].

Dual inhibitors have significant potential for tackling multiple stages of the life cycle of SARS-CoV-2. It can result in more potent antiviral effects and reduce the risk of viral resistance [13]. Certain alkaloids have demonstrated antiviral potential, making them viable candidates for treating COVID-19 [14]. In this study, we employed previously reported antiviral alkaloids to selectively target the two key proteins of SARS-CoV-2, namely the spike glycoprotein and main protease. The host cell surface receptor angiotensin-converting enzyme 2 (ACE-2) is recognized by the spike glycoprotein, which is present on the outer surface of coronaviruses [15]. S1 and S2 are the two functional units present in these spike glycoproteins. S1 has an N-terminal peptidase domain and a collectrine domain at the C-terminus. Spike glycoproteins bind to the N-terminal peptidase domain [16]. ACE 2 is the primary cellular receptor for coronavirus entry, which starts the infectious process [17]. The attachment of the host and viral cell membranes requires the interaction of the viral spike glycoprotein and host receptor binding domains, due to which viral nucleocapsids are transported into the host cell [18].

The main protease is the essential enzyme for the transcription and replication of viruses [19]. The main protease is responsible for the synthesis of non-structural proteins, which help viral proteins to come together. Thus, the viral transcription and replication can be stopped by targeting this protein [20]. The treatment of different viral infections such as HIV and HCV has often involved targeting proteases. Consequently, viral proteases are recognized as effective therapeutic targets as they are crucial to viral replication [21, 22].

Secondary metabolites from medicinal plants and microbes are widely used to discover new and advanced drugs for curing various diseases [23]. Since the 19^th^ century, after the discovery of secondary metabolites, many medicinally important alkaloids have been found. In the field of pharmacology, alkaloids are well-known secondary metabolites that show various bioactivities. More than 10,000 alkaloids have been found in various organisms, including plants, fungi and bacteria. Some alkaloids are potential antivirals and can be used for the therapeutic agents against COVID-19 [24].

The current study was designed to repurpose the reported antiviral alkaloids having the capability to be used as drug targets for the treatment of COVID-19. An *in-house* library of 102 alkaloids with reported antiviral potential was prepared and screened against the two stated receptors to find the dual inhibitors. The compounds with the highest docking scores were subjected to molecular dynamic simulations to confirm the stability of the ligand’s interactions with the receptors. Downstream analysis pointed out six lead compounds as dual inhibitors of spike protein and main protease that are supposed to be experimentally validated for potential drugs against this novel Corona virus-19.

## Material and methods

### Selection of ligands

Alkaloids, derived from plants and microbes, are prominent secondary metabolites with a history in drug discovery [23]. For this study, we collected 102 antiviral alkaloids, reported from various sources, through the literature survey (S1 Table).

### Ligands preparation

Structures of selected compounds were drawn using CS ChemDraw Pro version 6 (S1 Table). The Ligands database was produced through MOE (Molecular Operating Environment) version 2014 by applying default parameters. Energy minimization of the ligand database was performed for the stabilization of the ligands for docking [25].

### Target protein selection

**Fig 1:**
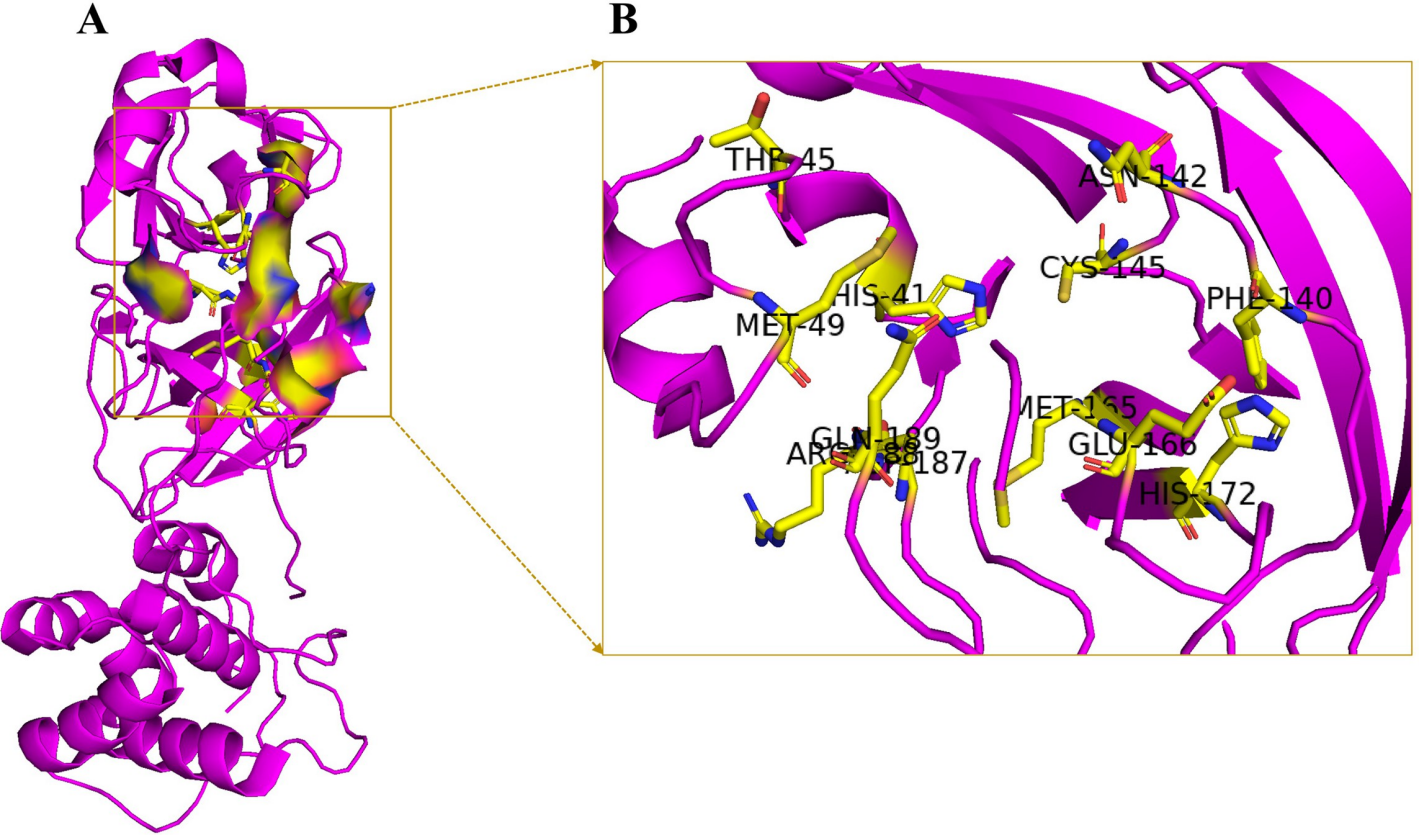
A) Prepared structure of 6LU7 with the selected active site highlighted in yellow surface B) Interacted key active site residues of the receptor.

**Fig 2:**
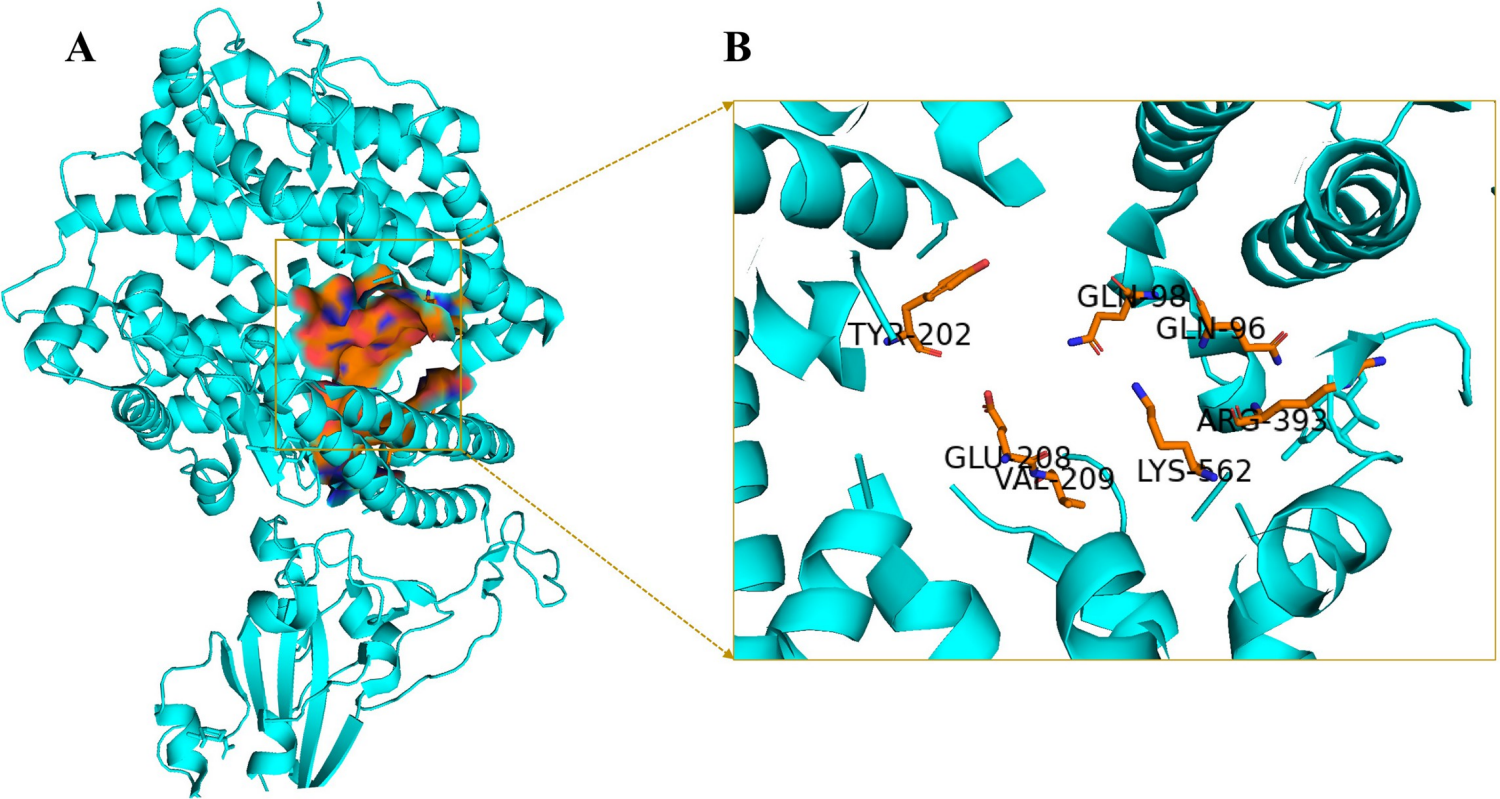
A) Prepared structure of 6LZG with the selected active site highlighted in orange surface B) Interacted key active site residues of the receptor.

Two target proteins of SARA-CoV-2 were selected to screen the antiviral alkaloids. These are the main proteases and spike receptor-binding domains in complex with its receptor, ACE2. The crystalline structures of the spike protein (ID: 6LZG) (Fig 1A) and main protease (ID: 6LU7) (Fig 2A) of SARA-CoV-2 were obtained from the PDB (protein data bank). The PDB structures of the proteins were selected on the basis of their crystallographic properties (S2 Table).

### Target proteins preparation

Both the selected target proteins were prepared in MOE by following steps;

#### Deletion of water molecules, repeated chains and, inhibitor

The three-dimensional structures of target proteins in PDB format were visualized in MOE. All the attached water molecules, non-unique chains, and co-crystalized inhibitors were deleted during the docking process. This was done to prevent impediment in the ligand and target protein’s interactions.

#### Protonation and energy minimization

The MOE software was used to perform 3D protonation and energy minimization with default settings. This procedure was carried out to fix any chain issues related to the target proteins.

#### Locating active site residues

The MOE Site Finder program was used to identify the active site for target proteins. The longest chain containing the reported key residues at the active site was selected. Dummy atoms were added on the active site to facilitate the docking process on the favorable active site residues.

### Docking process

Molecular docking of target proteins with a prepared ligand database was performed by computing the DOCK command in MOE software. During the docking process, the induced fit protocol was employed and the dummy atoms were used in the site module. Only dummy atoms were involved as protein active sites during the molecular docking process. Five conformational poses were retained in the result file. Ligand conformation with the highest docking score (S) was selected for protein-ligand interactions and downstream analysis.

### Validation of docking protocol

To ensure the precision and the dependence of the screening on docking, we employed a rigorous validation procedure based on redocking and superimposition techniques [26]. Initially, molnupiravir, the reference ligand, was docked into the specified receptor binding regions (RBD) of the spike protein (6LZG) using the MOE software. The resulting lowest energy pose of the molnupiravir-RBD complex was analyzed, where the characteristics of ligand-protein interactions and binding site were recorded. Subsequently, molnupiravir was separated from this complex and redocked, maintaining the same binding parameters. The final energy poses of both the original and redocked ligands were superimposed in PyMOL to determine the all-atom RMSD (root-mean-square deviation) value. An RMSD value of ≤2 Å or 0.2nm is typically accepted as a confirmation of a reliable docking method [26].

Furthermore, for the validation of the 6LU7 protein docking, the resulting 6LU7-N3 complex was superimposed with the PDB crystal structure 6LU7 that had the co-crystallized N3. This comparison provided further assurance regarding the docking protocol’s accuracy.

### MD simulation

The MD Simulation was conducted in explicit solvent using the Amber Package (version 18) [27]. For the complexes, the GAFF and ff99SB force fields were employed. To create a solvated system, a transferable intermolecular potential (TIP3P) water box was utilized, followed by the addition of sodium counterions to neutralize the system. To mitigate edge effects, periodic boundary conditions were applied. To ensure the accuracy of angles, bonds, and atom types, the docked protein complex underwent verification using the antechamber program. To achieve energy minimization, two methods were employed: 1000 steps of the conjugate gradient method, followed by 1500 steps of the steepest descent method, both executed at a threshold value of 0.9nm (9 Å). The molecular dynamics simulation was carried out in the NVT ensemble, employing the SHAKE algorithm to maintain bond constraints. Prior to simulation, the system underwent preprocessing through extrinsic heating and volume scaling over 0.2 ps, stabilizing the system at 300 K and 1 atm pressure. Temperature constancy was maintained using the Berendsen coupling integration algorithm. Throughout the MD simulation, atomic coordinates were recorded at intervals of 1.0 ps for trajectory files. Subsequent to the simulation, trajectory analysis was performed [28]. This encompassed calculations for parameters such as root mean square fluctuation (RMSF), root mean square deviation (RMSD), β-Factor, radius of gyration (Rg). Visualization of these results was accomplished using the Xmgrace software [29].

### Bioactivity score

The bioactivity score sums up a drug candidate’s potential to be a successful medication. Using the cheminformatics tool Molinspiration, the leads (six dual-active alkaloids) had their bioactivity scores verified. The bioactivity score of ligands for human receptors such as GPCRs, ion channels, nuclear receptors, kinases, proteases, and enzymes can be calculated using a free internet server [30].

### Drug-likeness and physiochemical properties

SwissADME server was used to investigate the physiochemical properties and drug-likeness of leads compounds. Drug-likelihood is determined through different rules and filters like Egan [31] Lipinski [32], Veber [33], Ghose [34], and bioavailability scores

### ADMET (pharmacokinetics) analysis

The drug’s ADMET (absorption, distribution, metabolism, excretion, and toxicity) features affect its pharmacokinetics. The ADMET characteristics of leads were assessed through the widely used and free web server pkCSM. The input files of the pkCSM server include the canonical SMILES of the selected ligands sourced from the PubChem database [35].

### PASS prediction

The top alkaloids were subjected to PASS (Prediction of Activity Spectra for Substances) analysis online server to find potential antiviral activities in addition to their predicted activities in this study. The PASS server is a tool created to predict the pharmacological effects, mechanisms of action, and biological roles of a molecule using structure-activity relationships of known chemical compounds. The predictions are presented as probabilities that indicate the likelihood of a compound being active (Pa) for a specific biological activity [36].

For the purposes of this study, we concentrated specifically on features with a Pa value larger than 0.7, which denotes a high probability that our top alkaloids will manifest that particular activity. In addition to this criterion, regardless of their Pa values, features directly relevant to antiviral activity were also chosen to emphasize the fundamental goal of identifying possible antiviral activities of our alkaloids.

## Results and discussion

### Active site prediction

The active site of 6LU7 is located around the amino acids His41 and Cys145 as the catalytic dyad in the S1 sub-pocket. These residues are important for the effective binding of ligand and the inhibition of hydrolytic activity of this protein. Glu166 is vital to keep it in active conformation [37]. Moreover, the binding to Thr45, Met49, Phe140, Met165, Glu166, Asp187, Asn142, His172, Arg188, and Gln189 causes the grid opening of the active site [38]. The selected active sites of 6LU7 included Thr25, Thr26, Leu27, His41, Val42, Cys44, Thr45, Ser46, Met49, Pro52, Tyr59, Phe140, Leu141, Asn142, Gly143, Ser144, Cys145, His163, His164, Met165, Glu166, Leu167, Pro168, His172, Asp187, Arg188, Gln189, and Thr190 (Fig 1B).

Among the predicted active sites, the selected active site of 6LZG included Leu5, His34, Gln96, Gln98, Ala99, Gln102, Tyr196, Tyr202, Trp203, Gly205, Asp206, Tyr207, Glu208, Val209, Arg219, Phe390, Leu391, Leu392, Arg393, Asn394, Ala396, Asn397, Glu398, Glu406, Val407 Ser511, Arg514, Lys562, Pro565, Trp566, and Lys582 (Fig 2B). Some of these residues were matched with the potential binding sites and favorable interacting residues of 6LZG [39, 40], while some residues were part of the SARS-cov-2 spike protein-ACE-2 interface [41].

### Ligand receptor interaction profile

For the main protease (6LU7), the docking scores of lead alkaloids were analyzed to assess their binding affinities. The top ten alkaloids, arranged in increasing order of binding affinities, were Isoliensinine, Neferine, Obaberine, Liensinine, Homoarmoline, Emetine, Norcycleanine, Fangchinoline, Thalrugosine, and Acrimarine F. The docking score range for these ligands varied from -7.7072 kcal/molto -5.4038 kcal/mol (S1 Table). Similarly, for the spike protein (6LZG), the docking scores of alkaloids were studied, revealing their respective binding affinities. The top-scoring ligands, ranked in increasing order of binding affinities, were Neferine, Isoliensinine, Liensinine, Acrimarine F, (+)-Stephibaberine, Emetine, Psychotrine, Fangchinoline, Aromoline, and Berbamine. The docking score range for these ligands ranged from -10.0245 kcal/mol to –5.8434 kcal/mol (S1 Table).

In the context of docking scores, a more negative value indicates a stronger binding affinity, signifying that the ligand forms a more stable complex with the protein. In molecular docking studies, all of the top dual active alkaloids showed docking scores that were higher than either of the two standards, molnupiravir with 6LZG and N3 with 6LU7. This highlights the potential of the alkaloids in the context of molecular affinity. Neferine demonstrated a particularly strong binding affinity, with a docking score of -7.5025 kcal/mol for 6LU7 and an exceptionally negative docking score of -10.0245 kcal/mol for 6LZG, suggesting a favorable interaction with both target proteins.

During molecular docking with 6LZG, three compounds, namely leurocristine, sophocarpine, and sophoridine, were found to be inactive, indicating no interaction with it. Similarly, two compounds, sophocarpine and sophoridine, demonstrated inactive behavior when docked with 6LZG, indicating no interaction with it.

For the N3 and 6LU7 interaction, N3 represented a docking score of -4.768 kcal/mol. Notable interactions included O11 and SG of Cys145; N12 and OG of Ser144 and O13 and NE2 of His163. The peptide like inhibitor, N3 is a helpful reference compound for the 6LU7 protein due to its well-known ability to block enzymes essential for viral replication [42]. The positive energy metrics and the significant interaction with Cys145 highlight the potential of this residue for protein functionality and inhibition [13, 37]. The interaction profile of N3 with 6LU7 is similar to the top dual active alkaloids in the library (S1A and S1B Fig).

According to the analysis, the docking score of molnupiravir with 6LZG was -5.275 kcal/mol. Notable interactions included the formation of a H bond between the molnupiravir atoms C23 and OE2 of Glu406 residue (B chain) and the strong interaction between the atoms O41 and NE2 of His34 residue (A chain). Antiviral potential of molnupiravir, notably against RNA viruses, have been the subject of substantial research [43]. A possible inhibitory effect on the 6LZG protein is suggested by its strong contact with His34, which is indicated by the considerable interaction energy (S1C and S1D Fig). Interestingly, none of the dual active alkaloids interacted with the residues similar to the interaction profile of the molnupiravir. This may give a novel mechanism of action for these alkaloids by pointing to a special form of binding and interaction. Such unique interactions may open up new treatment possibilities and shed light on their possible pharmacological advantages, necessitating more in-depth research to clarify their importance. The selection of molnupiravir and N3 as standards, supported by their recognized therapeutic relevance and noted interactions, offers confidence in the selection of lead compounds from the studied alkaloids.

### Dual inhibitors

Compounds with the potential to inhibit more than one target protein, that have an important role in a biological pathway are considered interesting leads in drug discovery experiments [44]. In this study, six compounds from the top ten lead alkaloids showed a dual inhibitory nature with the target proteins. These alkaloids are liensinine, neferine, isoliensinine, fangchinoline, emetine, and acrimarine F (Table 1).

**Table 1:** Interactions of the six lead ligands with 6LU7.

Sr. No.	Alkaloid Name	Docking Score (kcal/mol)	6LU7 interacting residues	Bond type	Bond Distance (nm)	Binding Energy (kcal/mol)	Hydrophobic interactions
1	Liensinine	-6.5842	Cys 145	H-donor	0.38	-1.0	Leu141, Ser144, His 41, Cys145, Phe140, Met165, Leu167, Pro168, Thr190, Glu166, Ala191, Asn14.
			Gln 189	π-H	0.4	-1.1	
2	Neferine	-7.5025	Asn 142	H-bond	0.25	/	Leu27, His41, Glu166, Leu16, Gly170, Pro168, Met165, Gln189, Leu141, Asn142, Ser46, Ser144, Cys145, Phe140, Gly143, Thr25, Met49, Thr26
			Glu 166	H-bond	0.23	/	
3	Isoliensinine	7.7072	Met 165	H-donor	0.43	-0.8	Gly143, Leu141, Thr26, His163, Ser46, Gln189, Pro168, His164, His41, Met49, Glu166, Ser144, Leu167, Cys145, Asn192.
4	Fangchinoline	-6.2187	Glu 166	H-donor	0.32	-1.0	Cys145, Gly143, Ser144, Asn142, Leu167, Met165, Gln189, Thr190
			Glu 166	π-H	0.43	-1.3	
			Glu 166	π-H	0.38	-0.8	
			Pro 168	π-H	0.4	-1.1	
5	Emetine	-6.2879	Glu166	π-H	0.45	-0.8	Pro168, Met165, Cys145, Ser144, Asn142, Gly143, Leu141, Gln189, Ala191, Phe140, Thr190, Leu50, Pro168, Met165, Cys145, Ser144, Asn142, Gly143, Leu141, Gln189, Ala191, Phe140, Thr190, Leu50, His164
			Asn142	H-bond	0.24	/	
			Gln189	H-bond	0.24	/	
6	Acrimarine F	-6.3713	Met49	π-H	0.39	-0.7	Phe140, Glu166, Leu141, Pro168, Leu167, Met165, Ala191, Ser144, Thr190, Cys145, Thr24, Ser46, Thr25, Thr26, Gly143, His41, Asn142, His164, Gln192
			Gln189	H-bond	0.21	/	
			Gln189	H-bond	0.17	/	
			Gln189	H-bond	0.34	/	
			Thr190	H-bond	0.28	/	

#### Liensinine

**Fig 3:**
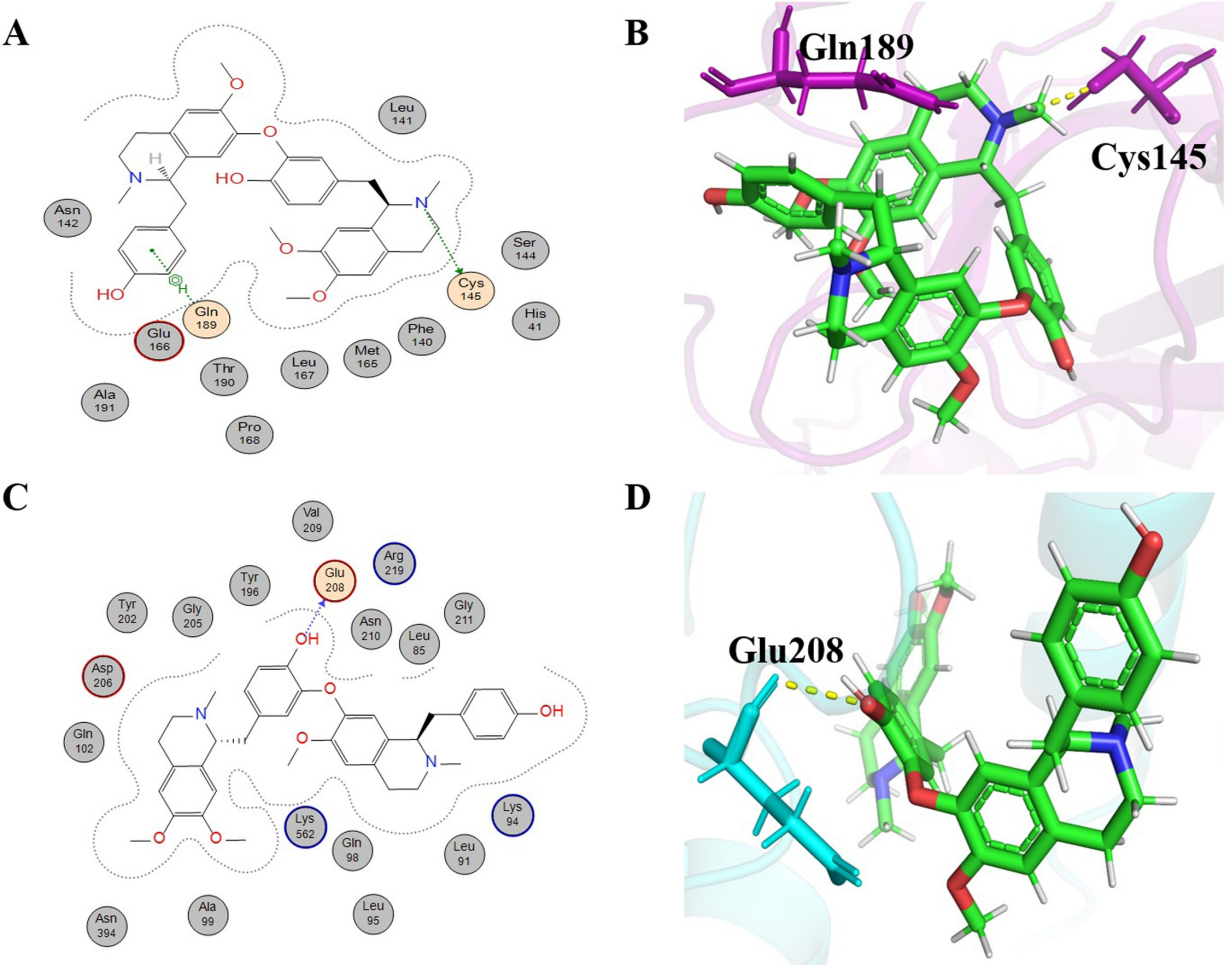
Schematic representation and 3D visualization of molecular interactions of Leinsinine with the target proteins. (**A)** Schematic depiction of interactions for liensinine with 6LU7, (**B**) 3D representation of liensinine (green) interactions with 6LU7 (purple) (**C)** Schematic depiction of interactions for Leinsinine with 6LZG and (**D**) 3D representation of Leinsinine (green) interactions with 6LZG (cyan) with the representation of hydrophobic (grey circles), hydrophilic interactions (orange circles) and hydrogen bonds (yellow dashed lines).

Liensinine (molecular formula: C_37_H_42_N_2_O_6_, IUPAC name: 4-[[(1R)-6,7-dimethoxy-2-methyl-3,4-dihydro-1H-isoquinolin-1-yl]methyl]-2-[[(1R)-1-[(4-hydroxyphenyl)methyl]-6-methoxy-2-methyl-3,4-dihydro-1H-isoquinolin-7-yl]oxy]phenol), is a bisbenzylisoquinoline alkaloid. It was extracted from the leaves and embryo of *Nelumbo nucifera* (Nymphaceae) [45]. Liensinine is an important alkaloid that has been reported to show different therapeutic importance, such as inhibition of autophagy and apoptosis enhancement in breast cancer cells, along with chemotherapeutic drugs [46], antiviral properties [45] and other important activities [47]. Liensinine, one of the lead alkaloids, gave -6.5842 kcal/mol binding energy to 6LU7 (Table 1). It formed two hydrogen bonds with the two key residues, the first one (H-donor type) with Cys145, one of the residues in the catalytic dyad of 6LU7, at a distance of 0.38 nm with -1.0 kcal/mol energy. It formed a second hydrogen bond (π-H) with Gln 189, which take part in active site grid opening, at a distance of 0.4 nm with -1.1 kcal/mol energy (Fig 3A and 3B). Residues that were involved in hydrophobic interaction were Leu141, Ser144, His 41, Cys145, Phe140, Met165, Leu167, Pro168, Thr190, Glu166, Ala191, and Asn14 (Table 1). The binding energy of liensinine with 6LZG was -8.7930 kcal/mol (Table 2). It formed a hydrogen bond (H-donor) with Glu208 at a distance of 0.28 nm with -1.1 kcal/mol (Fig 3C and 3D). Val209, Arg219, Asn210, Leu85, Gly211, Tyr196, Gly205, Tyr202, Asp206, Gln102, Asn394, Ala99, Lys562, Gln98, Leu95, Leu91, and Lys94 of the receptor (6LZG) were involved in hydrophobic interaction with liensinine (Table 2).

**Table 2:** Interactions of the six lead ligands with 6LZG.

Sr. No.	Alkaloid Name	Docking Score (kcal/mol)	6LZG interacting residues	Bond Type	Bond Distance (nm)	Binding Energy (kcal/mol)	Hydrophobic interaction
1	Liensinine	-8.7930	Glu208	H-donor	0.28	-1.1	Val209, Arg219, Asn210, Leu85, Gly211, Tyr196, Gly205, Tyr202, Asp206, Gln102, Asn394, Ala99, Lys562, Gln98, Leu95, Leu91, Lys94
2	Neferine	-10.0245	Gln98	π-H	0.37	-0.6	Tyr202, Gln102, Tyr196, Glu208, Val209, Asn210, Ala99, Trp566, Leu95, Pro565, Asp206, Gly395, Gly205, Leu391, Ala396, Asn394, Glu398, Asn397
			Gln198	H-bond	0.26	/	
			Asn210	H-bond	0.31	/	
			Lys562	π-cation	0.41	-0.9	
3	Isoliensinine	-9.6900	Val209	π-H	0.42	-0.8	Asn210, Leu91, Glu208, Trp203, Lys562, Gly205, Tyr202, Val212, Leu95, Pro565, Ala99, Tyr196, Asp206, Gln98, Asn397, Gly395, Gln102, Asn394
			Asn210	H-bond	0.27	/	
			Asn210	H-bond	0.24	/	
4	Fangchinoline	-7.9562	Gln98	π-H	0.38	-1.2	Gln102, Asp206, Glu208, Gly205, Arg219, Val209, Tyr196, Lys562, Pro565, Ala396, Trp566, Leu95, Val212, Ala99, Asn210
5	Emetine	-8.5419	Val209	π-H	0.42	-0.8	Arg219, Gln10, Gly205, Tyr196, Asn210, Glu208, Asn210, Val212, Leu91, Leu95, Gln98, Ala99, Leu391, Pro565, Lys582
6	Acrimarine F	-8.1630	Tyr202	H-π	0.47	-0.6	Arg219, Glu208, Tyr196, Leu95, Gln102, Lys562, Tyr203, Glu398, Arg514, Ser511, Asp206, Ala99, Gly205, Gln98, Asn210, Val209
			Asn210	H-bond	0.26		

#### Neferine

**Fig 4:**
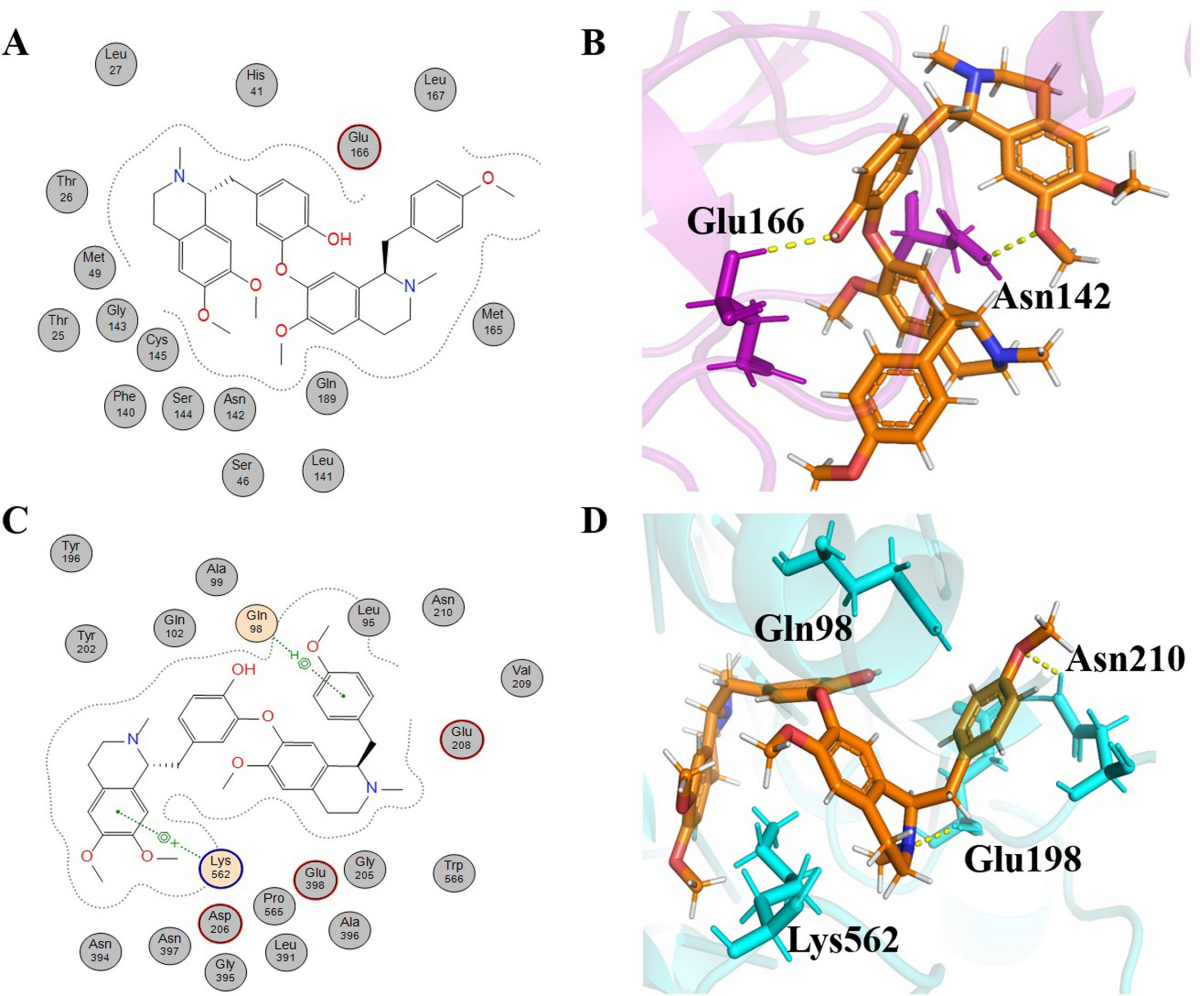
Schematic representations and 3D visualizations of molecular interactions of Neferine with the target proteins. (A) Schematic depiction of interactions for Neferine with 6LU7, (B) 3D representation of Neferine (orange) interactions with 6LU7 (purple) (C) Schematic depiction of interactions for Neferine with 6LZG and (D) 3D representation of Neferine (orange) interactions with 6LZG (cyan) with the representation of hydrophobic (grey circles), hydrophilic interactions (orange circles) and hydrogen bonds (yellow dashed lines).

Neferine is also a bisbenzylisoquinoline alkaloid like Liensinine (molecular formula: C_38_H_44_N_2_O_6_, IUPAC name: 4-[[(1*R*)-6,7-dimethoxy-2-methyl-3,4-dihydro-1*H*-isoquinolin-1-yl]methyl]-2-[[(1*R*)-6-methoxy-1-[(4-methoxyphenyl)methyl]-2-methyl-3,4-dihydro-1*H*-isoquinolin-7-yl]oxy]phenol) and extracted from the embryo and leaves of *Nelumbo nucifera* [45, 48]. Neferine has shown anti-HIV activity [45]. It has shown anti-hypertension activity in experimental rats [49]. Neferine showed -7.5025 kcal/mol binding energy with 6LU7. This alkaloid showed hydrophobic interaction with many key residues of the active site (Fig 4A and 4B) namely Leu27, His41, Glu166, Leu167, Gly170, Pro168, Met165, Gln189, Leu141, Asn142, Ser46, Ser144, Cys145, Phe140, Gly143, Thr25, Met49, and Thr26 (Table 1). MOE is customized for drug discovery and molecular modeling, using specific algorithms for docking predictions. In contrast, PyMOL is a molecular visualization tool emphasizing 3D structure visualization, which may depict different interaction details. These fundamental differences lead to varied interaction profiles when analyzing docked complexes. In the examination of neferine interactions using PyMOL, several interactions were identified that were not discernible in the MOE 2D schematic representation as shown in Fig 4. For the protein 6LU7, H bonding with Asn142 and Glu166 were observed, having bond distances of 0.25 and 0.23 nm, respectively. These interactions are significant for the metabolic activity of 6LU7 [38]. Neferine binding energy with 6LZG was -10.0245 kcal/mol with several hydrophobic interactions. Moreover, it exhibited four hydrogen bonds with amino acid residues of 6LZG (Table 2). It gave a π -H type interaction with the Gln98 residue of 6LZG at a distance of 0.37 nm with an energy of -0.6 kcal/cal. The second interaction of Neferine was of the π -cation type with the Lys562 residue of 6LZG at a distance of 0.41 nm with -0.9 kcal/mol energy. Two further H bond interactions were identified by PyMOL with residues Glu198 and Asn210, exhibiting bond distances of 0.26 and 0.31 nm, respectively (Fig 4C and 4D).

#### Isoliensinine

**Fig 5:**
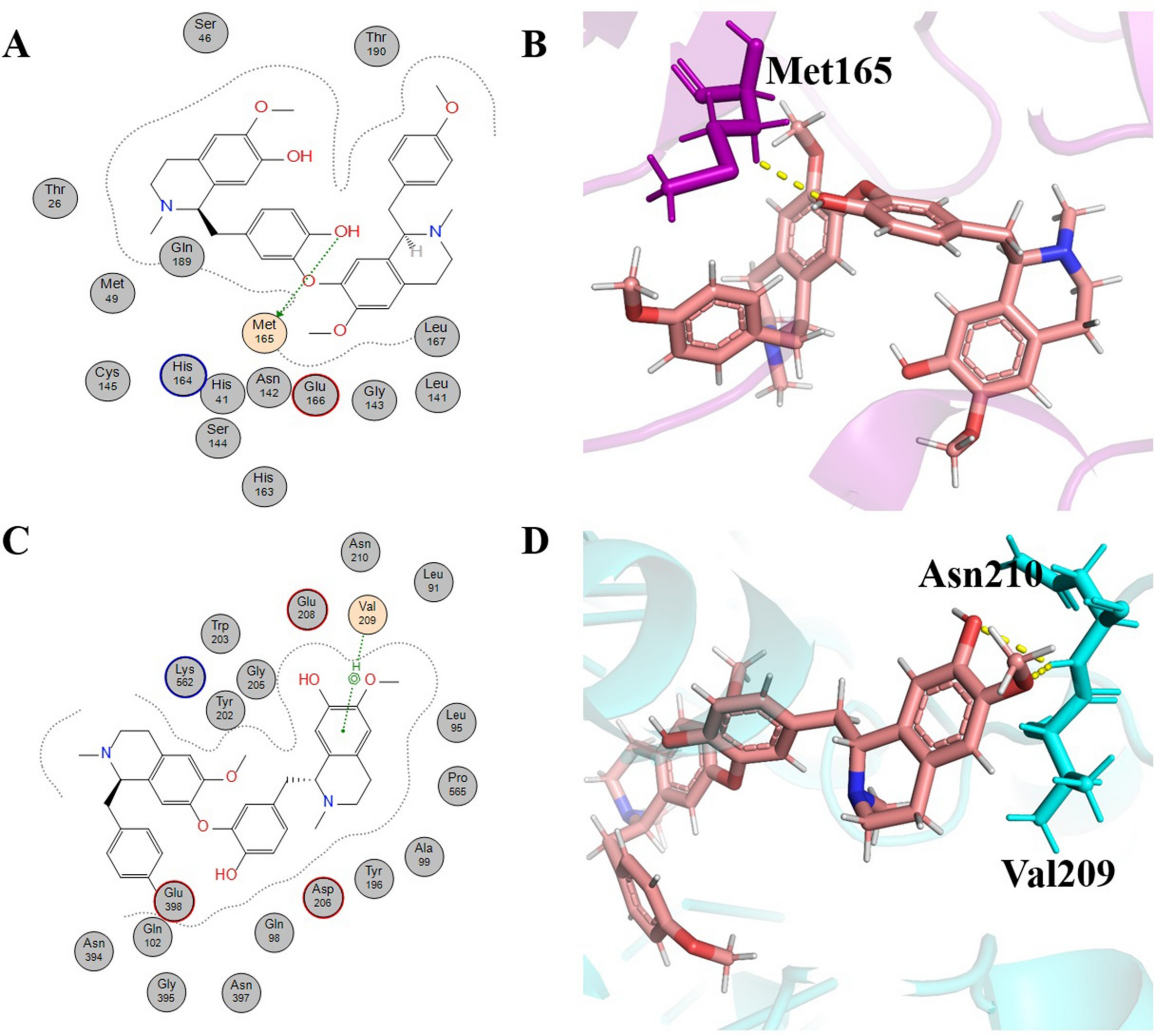
Schematic representations and 3D visualizations of molecular interactions of Isoliensinine with the target proteins. (A) Schematic depiction of interactions for Isoliensinine with 6LU7, (B) 3D representation of Isoliensinine (pink) interactions with 6LU7 (purple) (C) Schematic depiction of interactions for Isoliensinine with 6LZG and (D) 3D representation of Isoliensinine (pink) interactions with 6LZG (cyan) with the representation of hydrophobic (grey circles), hydrophilic interactions (orange circles) and hydrogen bonds (yellow dashed lines).

Isoliensinine is the third-highest-scoring compound that exhibited a dual inhibitory nature against COVID-19 protein targets. The IUPAC name of isoliensinine is (1R)-1-[[4-hydroxy-3-[[(1R)-6-methoxy-1-[(4-methoxyphenyl) methyl]-2-methyl-3,4-dihydro-1H-isoquinolin-7-yl]oxy]phenyl]methyl]-6-methoxy-2-methyl-3,4-dihydro-1H-isoquinolin-7-ol). It was initially extracted from *Nelumbo nucifera* and named "Lien Tze Hsin" by the Japanese researcher Ma-sao [50]. Isoliensinine has been reported to show respiratory system protection, neuroprotection, anti-type 2 diabetic activity, antioxidant, anti-HIV, and anti-cancer activity. This shows that liensinine is one of the important phytochemicals that has various medicinal properties [51]. In our analysis, isoliensinine showed -7.7072 kcal/mol binding energy with 6LU7 (Table 1). Isoliensinine exhibited both hydrophobic interaction and hydrogen bonding (Fig 5A and 5B). Residues of 6LU7 that were involved in hydrophobic interaction with isoliensinine were Gly143, Leu141, Thr26, His163, Ser46, Gln189, Pro168, His164, His41, Met49, Glu166, Ser144, Leu167, Cys145, and Asn192. The residue of receptor molecule (6LU7) that was involved in hydrogen bonding with isoliensinine was Met165. Met165 exhibited H-donor type interaction with isoliensinine at a distance of 0.43 nm with -0.8 kcal/mol energy (Table 1). That interaction could ease the binding of isoliensinine with 6LU7 by opening the active site grid [38]. Isoliensinine gave -9.6900 kcal/mol binding energy with 6LZG (Table 2). The Val209 residue of the receptor molecule (6LZG) formed hydrogen bonding of the π-H type with isoliensinine at a distance of 0.42 nm with -0.8 kcal/mol energy. By using PyMOL, the isoliensinine interaction profile exhibited two more H bonds with the 6LZG protein involving the Asn210 residue with the distances of 0.27 nm and 0.24 nm. Asn210, Leu91, Glu208, Trp203, Lys562, Gly205, Tyr202, Val212, Leu95, Pro565, Ala99, Tyr196, Asp206, Gln98, Asn397, Gly395, Gln102, and Asn394 were residues of 6LZG that took part in hydrophobic interaction with isoliensinine (Fig 5C and 5D).

The aforementioned dual-active alkaloids (Neferine, Liensinine, and Isoliensinine) belong to *Nelumbo nucifera*, commonly known as the lotus plant. They have various medicinal properties and are used in traditional Korean, Japanese, Indian, Thai, Oriental, Folk, and Chinese medicines [52, 53]. Along with other medicinal properties, this plant has antiviral activity against influenza virus [54], anti-HIV activity [45], and herpes simplex virus 1 [55]. Our results demonstrated the effectiveness of this plant and its phytochemicals against the novel Corona virus.

#### Fangchinoline

**Fig 6:**
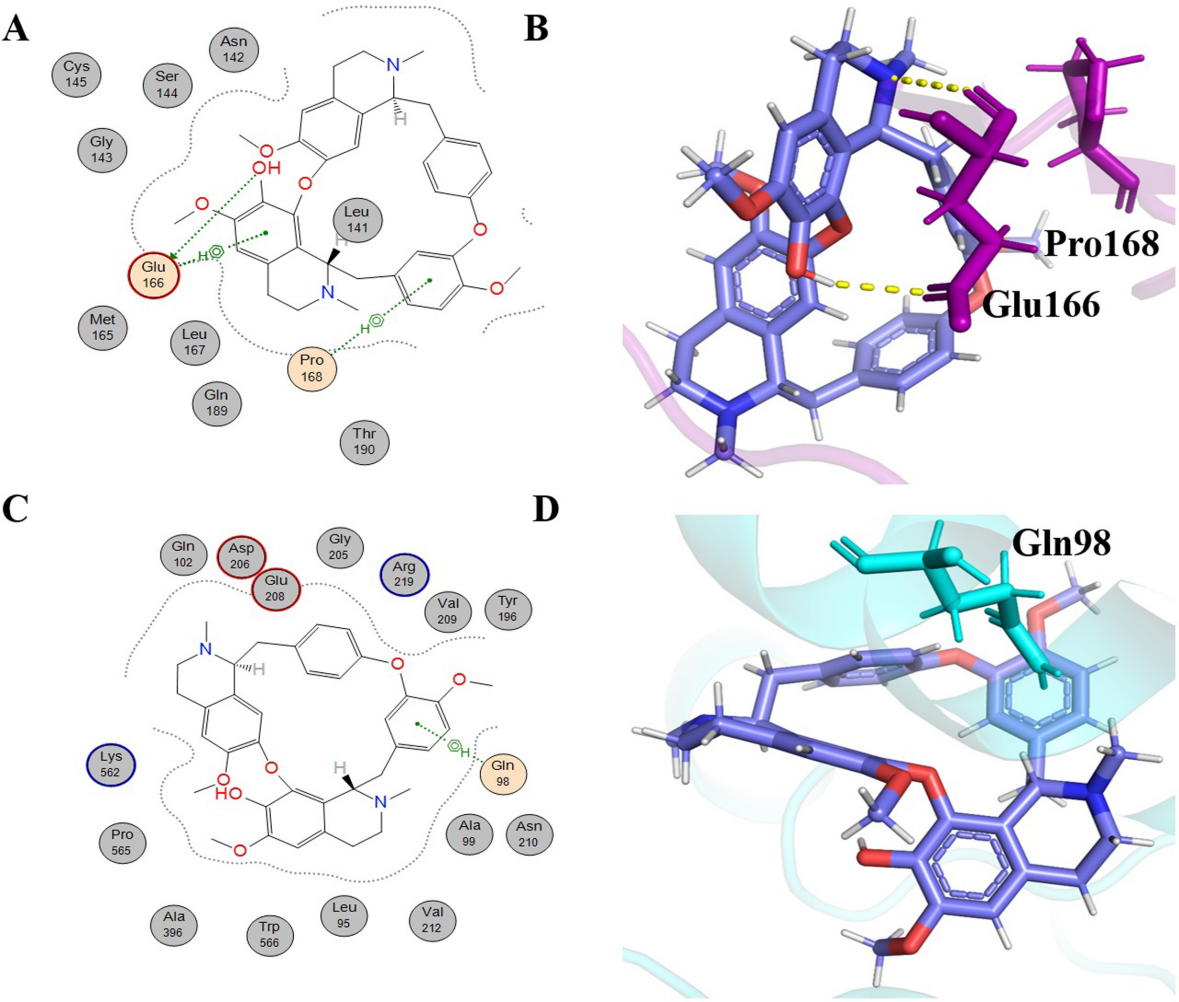
Schematic representations and 3D visualizations of molecular interactions of Fangchinoline with the target proteins. (A) Schematic depiction of interactions for Fangchinoline with 6LU7, (B) 3D representation of Fangchinoline (blue) interactions with 6LU7 (purple) (C) Schematic depiction of interactions for Fangchinoline with 6LZG and (D) 3D representation of Fangchinoline (blue) interactions with 6LZG (cyan) with the representation of hydrophobic (grey circles), hydrophilic interactions (orange circles) and hydrogen bonds (yellow dashed lines).

Fangchinoline belongs to the bisbenzylisoquinoline alkaloids. It is one of the active alkaloids isolated from the roots of *Stephania tetrandra* [56]. The IUPAC name and molecular formula of fangchinoline are (1*S*, 14*S*)-9,20,25-trimethoxy-15,30-dimethyl-7,23-dioxa-15,30 diazaheptacyclo[22.6.2.23,6.18,12.114,18.027,31.022,33]hexatriaconta-3(36),4,6(35),8,10,12(34),18,20,22(33),24,26,31-dodecaen-21-ol and C_37_H_40_N_2_O_6_, respectively. Previously, this compound has shown different types of activities, such as anti-inflammatory [57], anti-diabetic [58], anti-cancer [59], anti-oxidant effects [60], and anti-HIV activity [61]. In our results, Fangchinoline gave -6.2187 kcal/mol and -7.9562 kcal/mol binding energies with 6LU7 and 6LZG, respectively (Tables 1 and 2). Cys145, Gly143, Ser144, Asn142, Leu167, Met165, Gln189, and Thr190 were the residues of 6LU7 that were involved in hydrophobic interaction with fangchinoline (Table 1). Similarly, Glu166 of 6LU7 formed three hydrogen bonds with fangchinoline. These hydrogen bonds were of two types, i.e., H-donor and π-H. Glu166 interacts with fangchinoline by hydrogen bonding (H-donor type) at a distance of 0.32 nm with -1.0 kcal/mol. Glu166, which is important to keep 6LU7 in active confirmation, interacted with fangchinoline by π-H type interaction at the distances of 0.43 nm and energy of -1.3 kcal/mol. It depicted a hydrogen bond with the distance and energy of 0.38 nm with -1.3 kcal/mol and -0.8 kcal/mol energy, respectively. Pro 168 formed hydrogen bonding (π-H type) with fangchinoline with a distance of 0.40 nm at -1.1 kcal/mol energy. In addition to the hydrogen bond identified with Glu166 in the 2D schematic diagram, a subsequent analysis using PyMOL revealed an additional H bond involving Glu166, with a bond distance of 0.3nm (Fig 6A and 6B). The residues of 6LZG that are involved in hydrophobic interaction are Gln102, Asp206, Glu208, Gly205, Arg219, Val209, Tyr196, Lys562, Pro565, Ala396, Trp566, Leu95, Val212, Ala99, and Asn210 (Table 2). Gln98 of the receptor molecule (6LZG) showed a hydrogen bond (π-H interaction) with fangchinoline at a distance of 0.38 nm with -1.2 kcal/mol energy (Fig 6C and 6D). Among these residues, Gln98 is significant in the binding of 6LZG with the ACE-2 receptor. Binding of isoliensinine with Gln98 can restrict ACE-2 binding with 6LZG [41]. The multiple interactions of this ligand with the receptors indicated stable complex formation.

It is noteworthy that the chemical scaffold of these top dual inhibiting compounds namely liensinine, neferine, isoliensinine and fangchinoline, is bisbenzylisoquinoline alkaloid. The SARS-CoV-2 virus has been found as being susceptible to the bisbenzylisoquinoline alkaloids. According to certain studies, these alkaloids may have therapeutic advantages in the treatment or prevention of viral infections. Alkaloids and other vascular plant-derived substances are specifically suggested as COVID-19 therapy options [62]. An additional study has focused on natural bisbenzylisoquinoline alkaloids including Tetrandrine, Fangchinoline, and Cepharanthine as potential natural antiviral drugs against specific human coronaviruses [63].

#### Emetine

**Fig 7:**
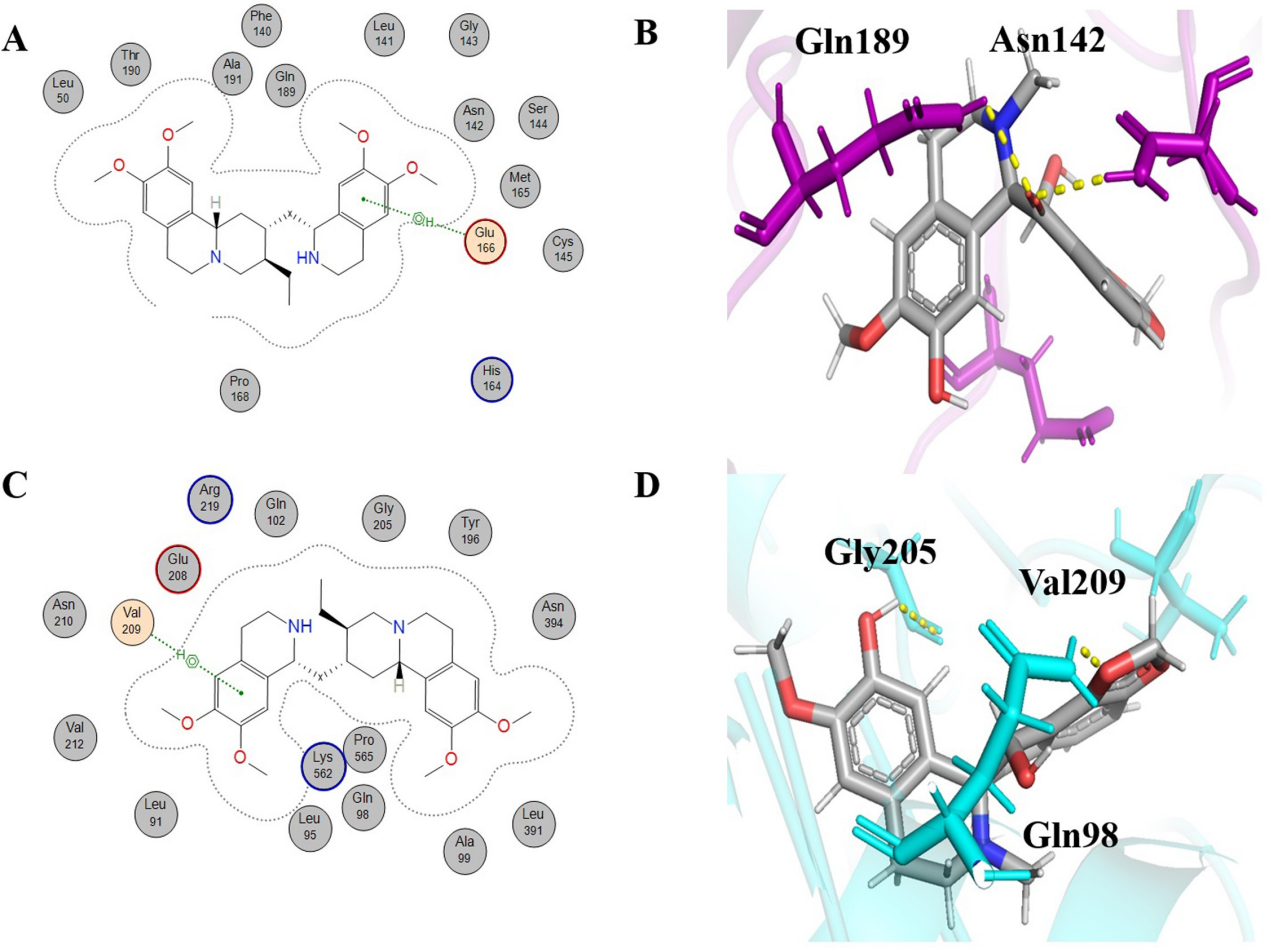
Schematic representations and 3D visualizations of molecular interactions of Emetine with the target proteins. (A) Schematic depiction of interactions for Emetine with 6LU7, (B) 3D representation of Emetine (grey) interactions with 6LU7 (purple) (C) Schematic depiction of interactions for Emetine with 6LZG and (D) 3D representation of Emetine (grey) interactions with 6LZG (cyan) with the representation of hydrophobic (grey circles), hydrophilic interactions (orange circles) and hydrogen bonds (yellow dashed lines).

Emetine (IUPAC name: (2S,3R,11bS)-2-[[(1R)-6,7-dimethoxy-1,2,3,4-tetrahydroisoquinolin-1-yl]methyl]-3-ethyl-9,10-dimethoxy-2,3,4,6,7,11b-hexahydro-1H-benzo[a]quinolizine) is an important alkaloid that has shown activity against viruses like herpes simplex virus, rift valley fever virus, echovirus, human metapneumovirus, and HIV-1 [64–66]. Emetine also exhibited anticancer, anti-parasitic, and anticontraceptive activity [67] and anti-malarial activity [68]. Emetine has been reported to be a potential antiviral agent against coronaviruses [69, 70]. Emetine (molecular formula: C_29_H_40_N_2_O_4_) is a natural alkaloid that is indigenous to Brazil. Three plant groups, including Alangiaceae, Icacinaceae, and Rubiaceae, contain emetine and its analogs [67]. Emetine gave -5.9981 kcal/mol binding energy with 6LU7 (Table 1). Residues of 6LU7 involved in hydrophobic interaction with emetine are Pro168, Met165, Cys145, Ser144, Asn142, Gly143, Leu141, Gln189, Ala191, Phe140, Thr190, Leu50, and His164. GLU166, which is important to keep 6LU7 in active confirmation, is involved in the formation of hydrogen bonding with emetine. Glu166 exhibited a π-H interaction with emetine at a distance of 0.45 nm with -0.8 kcal/mol energy. In addition to the bonds identified in the 2D schematic diagram, a subsequent analysis using PyMOL revealed two other hydrogen bonds with Asn142 and Gln189 both with the same Oxygen and same distance of 0.2nm (Fig 7A and 7B). It gave -6.9185 kcal/mol binding energy with 6LZG (Table 2). Arg219, Gln10, Gly205, Tyr196, Asn210, Glu208, Asn210, Val212, Leu91, Leu95, Gln98, Ala99, Leu391, Pro565 and Lys582 are the residues of 6LZG that are involved in hydrophobic interaction with emetine. Val209 residue of 6LZG Involved in Π-H interaction with emetine at a distance of 0.42 nm with -0.8 kcal/mol energy. In the PyMOL interaction analysis two more hydrogen bonds were observed with Gly205 and Gln298 with the bond distance of 0.27 nm and 0.2 nm respectively (Fig 7C and 7D).

#### Acrimarine F

**Fig 8:**
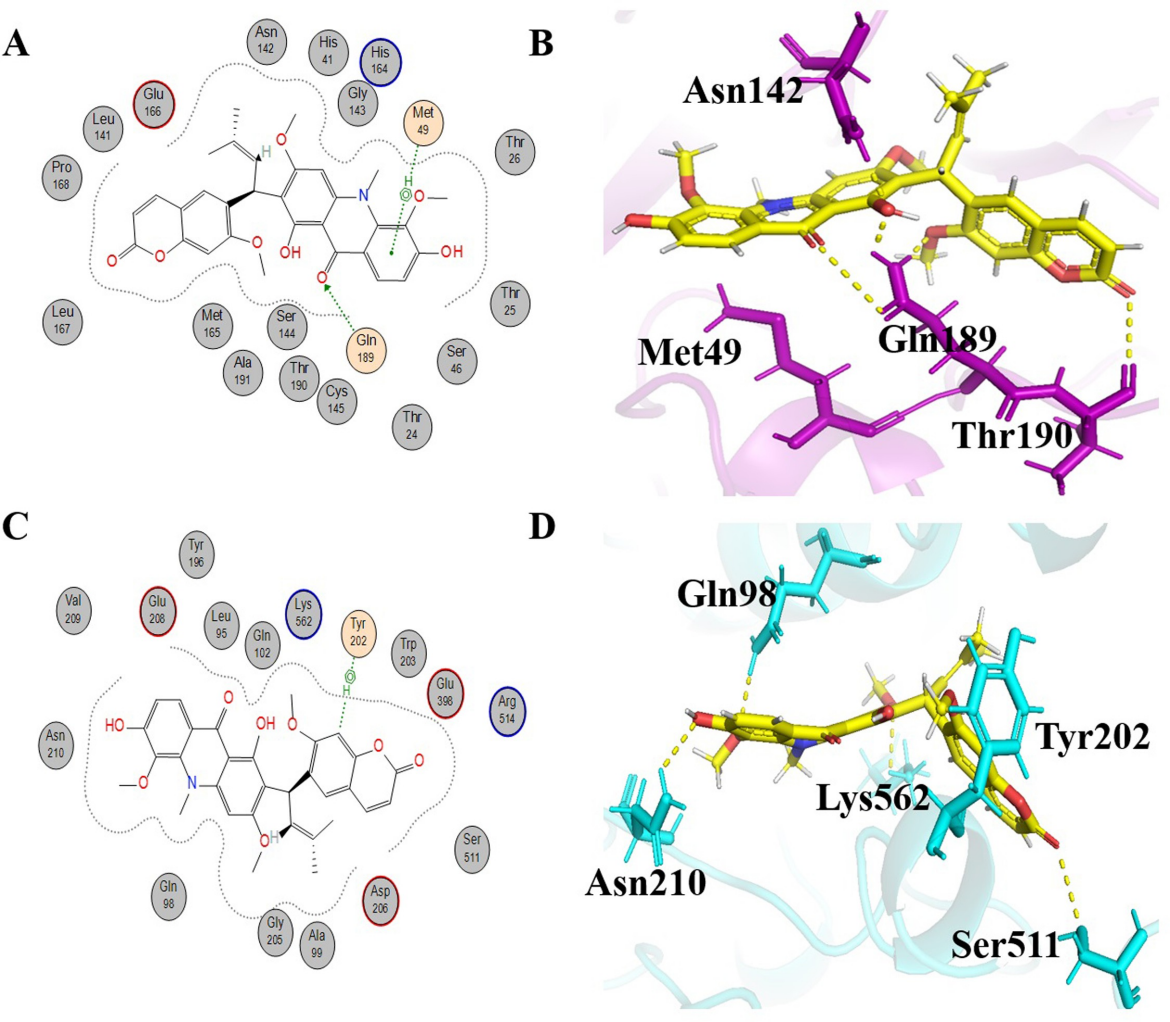
Schematic representations and 3D visualizations of molecular interactions of Acrimarine F with the target proteins. (A) Schematic depiction of interactions for Acrimarine F with 6LU7, (B) 3D representation of Acrimarine F (yellow) interactions with 6LU7 (purple) (C) Schematic depiction of interactions for Acrimarine F with 6LZG and (D) 3D representation of Acrimarine F (yellow) interactions with 6LZG (cyan) with the representation of hydrophobic (grey circles), hydrophilic interactions (orange circles) and hydrogen bonds (yellow dashed lines).

Acrimarine F, an important phytochemical belonging to alkaloids, has a molecular formula of C_31_H_29_NO_8_ and an IUPAC name of 1,6-dihydroxy-3,5-dimethoxy-2-[1-(7-methoxy-2-oxochromen-6-yl)-3-methylbut-2-enyl]-10-methylacridin-9-one, respectively. Acrimarine F shows -5.4310 kcal/mol and -6.9579 kcal/mol binding energies with 6LU7 and 6LZG, respectively. Acrimarine F shows hydrophobic interaction with a number of residues of 6LU7 and 6LZG (Tables 1 and 2). Gln 189 formed hydrogen bonding (H-acceptor type of interaction) at a distance of 0.33 nm with -0.8 kcal/mol energy. Met 49 gave a π-H type interaction with acrimarine F at a distance of 0.39 nm with -0.7 kcal/mol energy (Fig 8C and 8D). A total of five H bonds were observed between acrimarine F and 6LU7 protein. One of these bonds, associated with Gln189, was consistent with the representation in the 2D interaction scheme. Intriguingly, two additional H bonds with Gln189 were detected, exhibiting bond distances of 0.21 nm, 0.17 nm, and 0.34 nm, respectively. Furthermore, a H bonding involving the adjacent residue, Thr190, was also noted with the distance of 0.28nm. The last and fifth H bond was observed with Asn142 with the distance of 0.21 nm (Fig 8A and 8B). Tyr202 of 6LZG formed an H-π interaction with acrimarine F at a distance of 0.47nm with -0.6 kcal/mol energy. In examining the interactions of acramine f with the 6LZG protein, four H bonds were identified. These interactions involved the residues Asn210, Gln98, Ser511, and Lys562, exhibiting bond distances of 0.26 nm, 0.23nm, 0.32nm, and 0.25nm, respectively (Fig 8C and 8D). Overall, significant interactions can be found between dual-active alkaloids and the residues Cys145, Met 165, Glu166, Pro 168, and Met49, which are directly involved in the catalytic mechanism of 6LU7 [71], and significant hydrophobic interactions can be found with potential binding residues of 6LZG [39, 40].

### Docking protocol validation

The accuracy and precision of ligand location within the target protein structure are mandatory in molecular docking. The RMSD is a crucial indicator used to evaluate this accuracy [26]. The average distance between atoms in superimposed molecules is quantified by RMSD, which also provides information on how comparable the anticipated (docked) and known reference structures are. The trustworthiness of the docking process is typically demonstrated by a RMSD value closer to zero, which denotes that the docked pose resembles closely with the reference structure.

**Fig 9:**
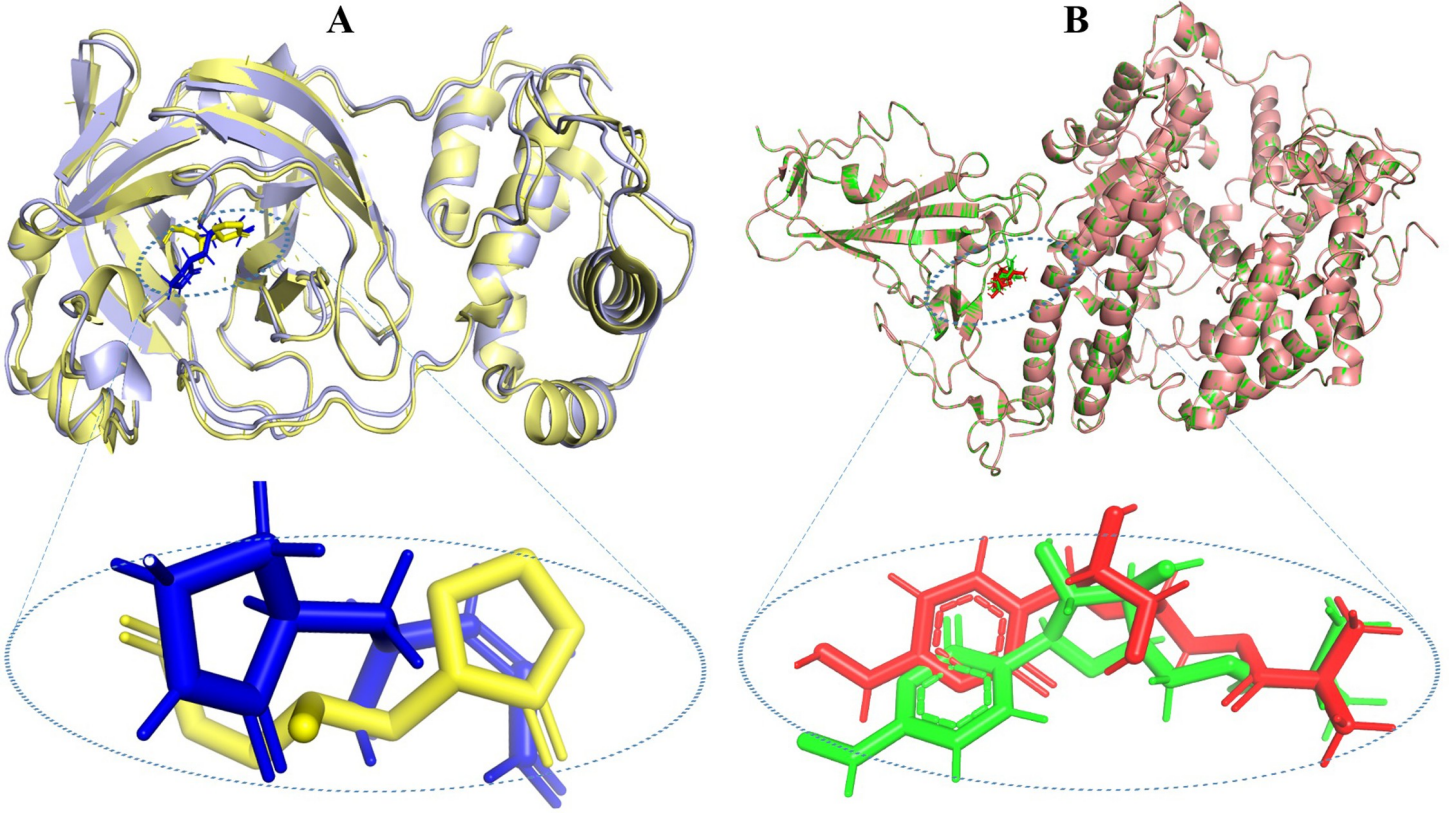
Docking validation through superimposition of complexes. (A) Superimposition of 6lu7 with co-crystallized ligand N3 (yellow) and docked N3 (blue) and (B) Superimposition of 6lzg protein with docked (red) and redocked (green) malnupiravir.

In the superimposition analysis of the 6LU7 complex with N3, there was an initial comparison between 306 and 309 atoms. However, during the refinement process, a series of alignment cycles rejected a few atoms, resulting in a final alignment between 284 atoms from each structure. The final RMSD value obtained was 0.725 Å, indicating a very close alignment between the docked and reference structures, albeit with some minor deviations (Fig 9A). In the subsequent study, the complex of 6LZG with molnupiravir was examined. Out of the total 791 atoms involved in both structures, the superimposition resulted in an RMSD value of 0.000 Å (Fig 9B), signifying an almost identical alignment between the docked and re-docked structures. Collectively, these RMSD values emphasize the precision of the docking methodologies, as the docked ligands closely align with their respective reference structures. We may conclude that the same technique will probably provide accurate poses for alkaloids in the library received from the literature survey by showing that our docking protocol can reproduce known poses of reference compounds with such precision.

### Dynamic behavior of lead compounds

The MD simulations explored the dynamic behavior of the COVID-19 main protease crystal structure complexed with the N3 inhibitor (6LU7). In the trajectory analysis, the drugability potential of the ligands acrimarine-f, fangchinoline, and liensinine was evaluated. Four key physical properties, namely root mean square deviation (RMSD), β-factor, root mean square fluctuation (RMSF), and radius of gyration (Rg), were plotted for each ligand.

**Fig 10:**
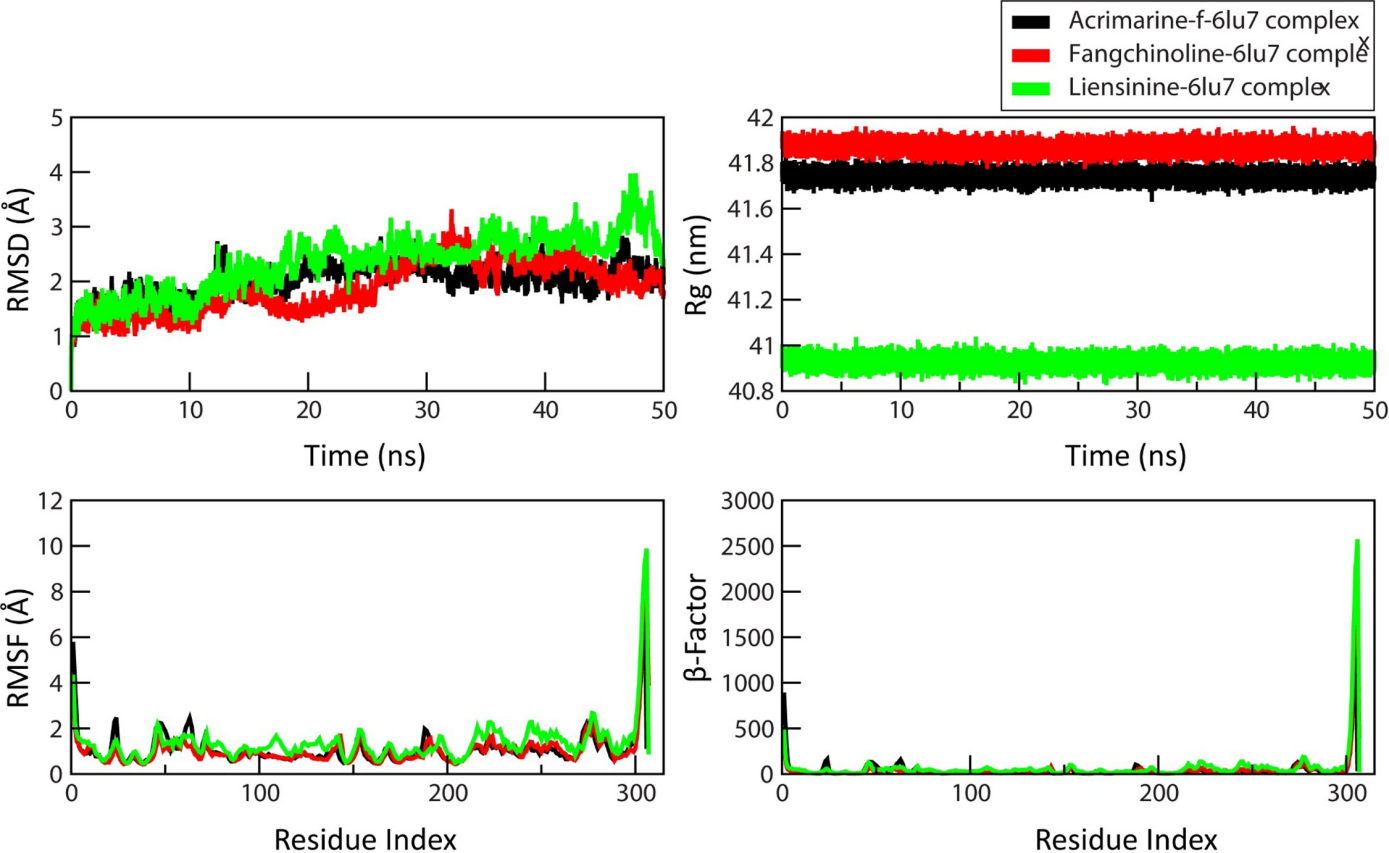
The Trajectory analysis of COVID-19 main protease protein (6LU7) in complex with ligands Acrimarine-f (black), Fangchinline (red) and Liensinine (green) at 0 ns and 50 ns of MD simulations under 300K thermal condition, (A) RMSD (B) RMSF (C) Radius of Gyration (D) ß-factor.

RMSD, a measure of structural rigidity, provided information about the structural stability of complexes over a 50 ns time scale. Notably, the liensinine-6LU7 complex displayed more significant structural disorientation compared to the other complexes (Fig 10). This structural change became pronounced between 45 ns and 50-ns (S2 Fig). While minor geometric shifts were observed in the liensinine-6LU7 complex due to ligand binding, a comprehensive comparison of RMSD values across all complexes showcased noticeable differences. RMSF, an indicator of atomic fluctuations, highlighted flexible and rigid regions within the drug-target complexes. The graph revealed a peak in RMSF at the protein’s C-terminal region, indicating higher positional displacement. The mobility of specific residues, including CYS-300, SER-301, GLY-302, VAL-303, THR-304, PHE-305, and GLN-306, was validated by β-factor analysis. These residues predominantly reside in the loop region, except for CYS-300 and SER-301, which are helix-terminating residues. The increase in the radius of gyration (Rg) for acrimarine-f-6LU7 and fangchinoline-6LU7 complexes suggested reduced protein compactness and, consequently, decreased stability. However, the liensinine-6LU7 complex exhibited a smaller Rg, indicative of enhanced stability.

**Fig 11:**
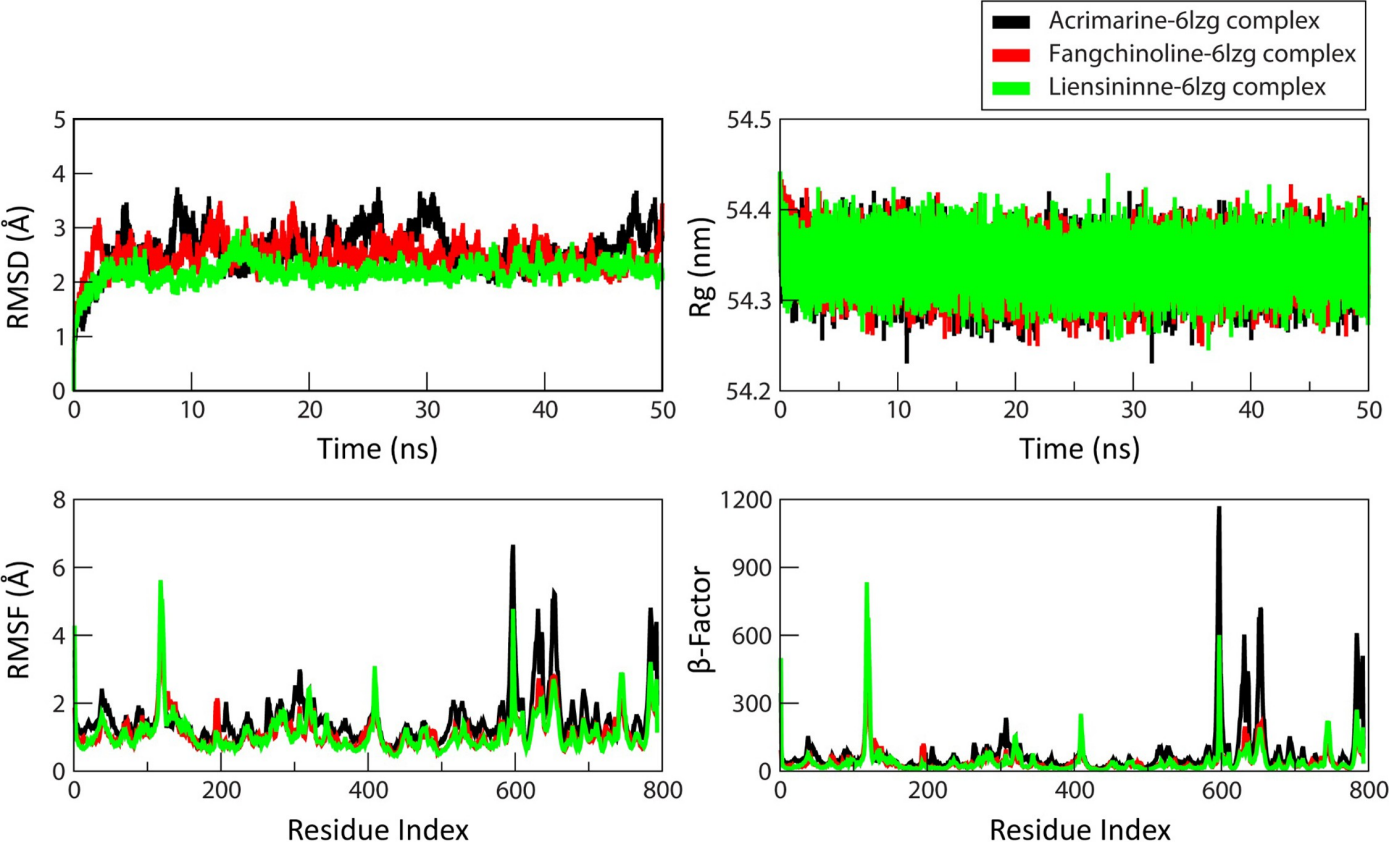
The Trajectory analysis of Coronavirus spike receptor-binding domain (6LZG) in complex with ligands Acrimarine-f (black), Fangchinline (red) and Liensinine (green) at 0 ns and 50 ns of MD simulations under 300K thermal condition, (A) RMSD (B) RMSF (C) Radius of Gyration (D) ß-factor.

For the ligands acrimarine-f, fangchinoline, and liensinine, MD simulation and trajectory analysis were performed to assess their interaction with the Coronavirus spike receptor-binding domain (6LZG). Throughout the simulation, observable variations in RMSD were seen in the acrimarine-f-6LZG, fangchinoline-6LZG, and liensinine-6LZG complexes. The average RMSD of 13.7 indicated continuous deviations in the overall protein structure, with acrimarine-f-6LZG displaying a relatively higher RMSD value (Figs 11 and S3). Examining the protein-ligand complexes’ Rg values revealed minimal impact on the 6LZG protein structure due to ligand binding. The binding residues demonstrated consistent behavior, as reflected by constant RMSF values. Structural shifts near residues 117, 409, 597, 748, and 784 were attributed to the terminal residues within the 6LZG protein, a conclusion supported by β-factor analysis.

### Bioactivity score of lead compounds

The majority of medications work pharmacologically by attaching to biological targets like ion channels, receptors, and enzymes. These targets are frequently the focus when developing new therapeutic drugs since they are essential in influencing physiological processes. A molecule’s potential effectiveness is evaluated quantitatively using a bioactivity score based on how it is expected to interact with certain biological targets. This rating essentially acts as a predictor, showing how a molecule may affect the way receptors, enzymes, or ion channels function. This method offers significant knowledge into the underlying molecular interactions for upcoming research decisions and prospective drug development in addition to prioritizing desirable compounds [33].

If the bioactivity score is more than 0, the substance is thought to be biologically more active. Compounds with a moderate level of activity have bioactivity scores between -0.50 and 0.0. If the bioactivity score is less than 0.50, the chemicals are regarded as inactive [33]. Bioactivity score analysis of the six lead compounds revealed values in the range between 0.0 and -0.50, indicating they can effectively interact with their respective biological targets and are predictive to have potential therapeutic effects as anti-COVID drug. Emetine and acrimarine F exhibited better bioactivity scores compared to other bioactive alkaloids (S3 Table).

### Drug-likeness and physiochemical properties

Six leads (dual-active alkaloids) were subjected to drug-likeness and physiochemical analysis through the Swiss ADME. Drug-likeness, physiochemical properties and ADMET properties, are closely associated with each other [2, 72]. Five rules make up the drug-likeness analysis in the Swiss ADME online software: Veber’s rule, Lipinski’s rule, Ghose’s rule, Muegge’s rule, and Egan’s rule. All the leads followed Lipinski’s rule with the exception of their molecular weight exceeding the typical threshold. Furthermore, all leads followed the Veber and Egan rules, but none of them followed the Ghose rule (Table 3). This indicates the structural resemblance of lead alkaloids with the ideal drugs [73]. The satisfactory adherence of lead alkaloids to drug likeness rules reflects their physicochemical properties falling within the acceptable range (Table 4). This correlation is of great importance as it enhances the applicability of lead alkaloids for potential drug development.

**Table 3:** Drug-likeness evaluation of the six dual-active alkaloids.

Sr. No.	Compound name	Lipinski Rule	Ghose Rule	Veber Rule	Egan Rule	Bioavailability score
1	Liensinine	Yes	No	Yes	Yes	0.55
2	Neferine	Yes	No	Yes	Yes	0.55
3	Isoliensinin	Yes	No	Yes	Yes	0.55
4	Fangchinoline	Yes	No	Yes	Yes	0.55
5	Emetine	Yes	No	Yes	Yes	0.55
6	Acrimarine F	Yes	No	Yes	Yes	0.55

**Table 4:** Physiochemical properties of six dual-active alkaloids.

Sr. No.	Alkaloid	M. wt	Log P	nRB	nHBA	nHBD	TPSA	MR
1.	Liensinine	610.74	5.17	9	8	2	83.86	183.55
2.	Neferine	624.77	5.47	10	8	1	72.86	188.02
3.	Isoliensinine	610.74	5.16	9	8	2	83.86	183.55
4.	Fangchinoline	608.72	5.14	3	8	1	72.86	181.60
5.	Emetine	480.64	4.24	7	6	1	52.19	147.05
6.	Acrimarine F	543.56	4.81	6	8	2	120.36	154.75

M. wt: molecular weight, nRB: number of rotatable bond, nHBA: no of hydrogen bond acceptor, nHBD: no of hydrogen bond donor, TPSA: Topological Polar Surface Area, and MR: molar refractivity

### ADMET analysis

Prior to a clinical trial, ADMET analysis is crucial for medication design and lowers the likelihood of a drug failure [35]. ADMET analysis is critical for determining the pharmacokinetics of leads. The online tool pkCSM was used to conduct the ADMET analysis. The following are comprehensive explanations of these six chemicals’ absorption, distribution, metabolism, excretion, and toxicity:

#### Absorption parameters analysis

Skin permeability, p-glycoprotein inhibitor, gastrointestinal absorption, aqueous solubility, p-glycoprotein substrate, and CaCO_2_ permeability are important parameters affecting a drug’s absorption [74]. The ***water solubility*** of the leads ranged from -3.485 to -3.886 log mol/L. Liensinine showed the highest water solubility (-3.485 log mol/L) while Fangchinoline showed the lowest water solubility (-3.886 log mol/L). There is a likelihood that all leads might serve as substrates for p-glycoproteins. Furthermore, there is a significant probability that they could function as inhibitors of both p-glycoprotein I and II (Table 5). *CaCO2 permeability* and *GI absorption* affect the drug’s bioavailability. A substance that has GI absorption > 30% exhibits enhanced body absorption. All selected compounds show good GI absorption, ranging from 95% to 87%. Acrimarine F showed the highest GI absorption (95%) (Table 5). All six leads show lower caco2 permeability ranges from 0.75 cm/s (Emetine) to 0.1220.75 cm/s (Acrimarine F). The selected drug candidates also showed admissible skin permeability (Table 5).

**Table 5:** ADMET properties of the six lead dual-active alkaloids.

Sr. No	Property	Model Name	Liensinine	Neferine	Isoliensinine	Fangchinoline	Emetine	Acrimarine F	Unit
1.	Absorption	Water solubility	-3.485	-3.882	-3.614	-3.886	-3.666	-3.725	Numeric (log mol/L)
		Caco2 permeability	0.561	0.368	0.548	0.43	0.75	0.122	Numeric (log Papp in 10^−6^ cm/s)
		Intestinal absorption	87.045	88.197	89.935	91.942	91.032	95.097	Numeric (%Absorbed)
		Skin Permeability	-2.735	-2.735	-2.735	-2.735	-2.798	-2.735	Numeric (log Kp)
		P-glycoprotein substrate	Yes	Yes	Yes	Yes	Yes	Yes	Categorical (Yes/No)
		P-glycoprotein I inhibitor	Yes	Yes	Yes	Yes	Yes	Yes	Categorical (Yes/No)
		P-glycoprotein II inhibitor	Yes	Yes	Yes	Yes	Yes	Yes	Categorical (Yes/No)
2.	Distribution	VDss (human)	-0.8	-0.542	-0.766	-0.81	1.596	-1.298	Numeric (log L/kg)
		Fraction unbound (human)	0.188	0.251	0.205	0.323	0.204	0.109	Numeric (Fu)
		BBB permeability	-1.046	-1.047	-1.039	-0.993	-0.394	-1.039	Numeric (log BB)
		CNS permeability	-2.57	-2.526	-2.568	-2.321	-2.067	-2.956	Numeric (log PS)
3.	Metabolism	CYP2D6 substrate	Yes	Yes	Yes	No	Yes	No	Categorical (Yes/No)
		CYP3A4 substrate	Yes	Yes	Yes	Yes	Yes	Yes	Categorical (Yes/No)
		CYP1A2 inhibitior	Yes	No	No	No	No	No	Categorical (Yes/No)
		CYP2C19 inhibitior	Yes	No	Yes	No	Yes	Yes	Categorical (Yes/No)
		CYP2C9 inhibitior	No	No	No	No	No	Yes	Categorical (Yes/No)
		CYP2D6 inhibitior	Yes	No	Yes	No	Yes	No	Categorical (Yes/No)
		CYP3A4 inhibitior	Yes	Yes	No	No	No	Yes	Categorical (Yes/No)
4.	Excretion	Total Clearance	0.87	1.01	0.99	0.704	0.993	0.847	Numeric (log ml/min/kg)
		Renal OCT2 substrate	No	No	No	No	No	No	Categorical (Yes/No)
5.	Toxicity	AMES toxicity	No	No	No	Yes	No	No	Categorical (Yes/No)
		Max. tolerated dose (human)	0.296	0.278	0.282	0.199	-0.019	0.186	Numeric (log mg/kg/day)
		hERG I inhibitor	No	N0	No	No	No	No	Categorical (Yes/No)
		hERG II inhibitor	Yes	Yes	Yes	Yes	Yes	Yes	Categorical (Yes/No)
		Oral Rat Acute Toxicity (LD50)	2.385	2.36	2.453	2.642	2.793	2.097	Numeric (mol/kg)
		Oral Rat Chronic Toxicity (LOAEL)	2.625	2.214	3.668	1.287	0.674	1.797	Numeric (log mg/kg_bw/day)
		Hepatotoxicity	Yes	No	Yes	No	No	Yes	Categorical (Yes/No)
		Skin sensitivity	No	No	No	No	No	No	Categorical (Yes/No)
		*T*.*Pyriformis* toxicity	0.285	0.285	0.285	0.285	0.327	0.285	Numeric (log ug/L)

#### Distribution parameters analysis

Parameters that affect drug distribution are the steady-state volume of distribution (VDss), central nervous system permeability, and the blood-brain barrier [75]. Drugs have a *VDss* value of >0.45 log L/kg. All leads showed a VDss value less than the admissible range except emetine. Emetine has a VDss value of 1.596 log L/kg. Also, Emetine, unlike the other five leads, can cross the *blood-brain barrier* (log PS: -0.394). *CNS permeability* is determined by the log PS value, which typically falls between >-2 and <-3 [2]. The observed range of log PS values, from -2.067 (emetine) to -2.956 (acrimarine F), suggests that the lead alkaloids exhibit varying levels of permeability across the blood-brain barrier (Table 5). Ligands with log PS values closer to -2 have a higher likelihood of crossing the barrier and accessing the CNS. On the other hand, ligands with log PS values closer to -3 may have reduced CNS permeability, making them less likely to reach the brain.

The unbound fraction of a medication affects its pharmacological and pharmacokinetic features which interacts with target proteins [76]. The *unbound fraction* of leads ranges from 0.323 Fu (for Fangchinoline) to 0.109 Fu (for Acrimarine F) (Table 5).

#### Metabolism Parameters analysis

Drug metabolism is significantly influenced by cytochrome p450 enzymes [77]. For the metabolic examination of leads, five distinct cytochrome p450 enzymes (CYP2D6, CYP2C9, CYP1A2, CYP3A4, and CYP2C19) were considered. One of the most significant members of the family of metabolic enzymes, which is involved in the metabolism of half of the medications now in use, is CYP3A4 [78]. All leads acted as substrates for CYP3A4. Similarly, they were CYP2D6 substrates, with the exception of fangchinoline and acrimarine F. Liensinine acts as an inhibitor for four cytochrome enzymes except CYP2C9. On the other hand, Neferine only inhibited CYP3A4. Isoliensinine and emetine acted as inhibitors for two CYPs, i.e., CYP2C19 and CYP2D6. Acrimarine F inhibits CYP3A4, CYP2C9, and CYP2C19. Fangchinoline is the only lead that does not act as an inhibitor for any of the CYP450 enzymes, which means its metabolism will not be interrupted by the action of any of the CYP450 enzymes (Table 5).

#### Excretion and toxicity parameters

The total clearance of the leads ranged from 1.01 log ml/min/kg (for Neferine) to 0.70 log ml/min/kg (for fangchinoline). OCT2 is a crucial transporter that is essential for the excretion of pharmaceuticals and endogenous substances through urine [79]. None of the bioactive compounds acted as a substrate for renal OCT2. Expectedly, these alkaloids can’t easily be excreted from the kidney because they are not acting as OCT2 substrates. These compounds might be excreted out through other routes [80]. None of the leads caused AMES toxicity or skin sensitivity except fangchinoline, which causes AMES toxicity. Neferine, fangchinoline, and emetine were non-hepatotoxic compounds, but liensinine, isoliensinine, and acrimarine F caused hepatotoxicity (Table 5). One of the crucial factors in determining the toxicity of potential substances is the hER gene, which has a direct relationship with cardiotoxicity. The hER gene inhibition causes potentially deadly heart rhythm disturbance. The hERG channel plays a pivotal role in cardiac repolarization. Its inhibition can lead to QT interval prolongation and potential cardiac issues. Such knowledge assists in designing safer compounds during drug discovery [81, 82]. All six selected alkaloids did not inhibit hERG I but showed an inhibitory effect toward hERG II. While these compounds target specific biological pathways due to hERG II inhibition, there will be fewer chances of cardiotoxicity during administration (Table 5). Chronic (LOAEL) and acute (LD50) toxicity analyses were performed to ensure compound safety when administered. Acrimarine has an acute toxicity value of 2.097 mol/kg, while emetine has one of 2.793 mol/kg. The highest and lowest chronic toxicity values were shown by isoliensinine (3.668 log mg/kg bw/day) and emetine (0.674 log mg/kg_bw/day), respectively. According to the results of acute LD50 toxicity analysis, emetine causes the lowest toxicity as compared to other selected alkaloids because the higher the value of LD50, lesser the toxicity, or vice versa [83, 84]. Similarly, according to LOAEL value, emetine is a safer alkaloid as a drug as compared to other lead alkaloids as it haa a higher LOAEL value (Table 5).

The strong binding affinity of these alkaloids with key viral targets suggests their potential to interfere with viral replication and infection. Their ability to inhibit p-glycoproteins could enhance drug efficacy by reducing efflux from cells. The favorable water solubility, gastrointestinal absorption, metabolism, and excretion profiles indicate good pharmacokinetic properties that are essential for effective drug delivery. Low toxicity and high LD50 values of these alkaloids, particularly for emetine and fangchinoline, make them promising antiviral drug candidates. Overall, these findings open up new doors for the development of targeted drugs with potential clinical uses and offer insightful information for designing therapeutic drugs against COVID-19.

### PASS parameters analysis

Based on their projected affinity values (Pa), some additional features of the investigated compounds were observed. It was clear that areas with Pa values higher than 0.7 were of the utmost importance. Liensinine, isoliensinine and neferine represented similar predicted biological activities due to their similar structural nature; bisbenzylisoquinoline alkaloid (S4 Fig). For example, these all may function as nicotinic alpha4beta4 receptor agonists. These neurotransmission-related receptors might not have any direct antiviral action. Modulating neurotransmission, however, might have indirect effects on the immune system or the body’s response to a viral infection. Also, its predicted antitussive property, which reduces coughing and provides symptomatic relief, can be quite helpful for viral respiratory infections. Additionally, their fibrinolytic properties, which help dissolve blood clots, can prevent clotting events occasionally found in viral infections (S4 Fig).

Fangchinoline exhibited a wide variety of characteristics (S5 Fig). Its capacity to stimulate histamine release has potential drawbacks. Although histamine is essential for immune responses, too much release might result in allergic reactions. The apoptosis agonist property of this substance also merits consideration; by encouraging planned cell death, it may be able to limit viral propagation within the body (S5 Fig).

We observed specific predictions for some alkaloids like Acramarine F. With Pa values of 0.744 and 0.728, Acramarine F was expected to be a CYP2A11 substrate as well as a spasmolytic (S6 Fig). A compound’s metabolism can affect its safety and efficacy and while being a CYP2A11 substrate might not directly correspond with antiviral benefits, the spasmolytic nature can be helpful for viral infections that cause muscular spasms. Acramarine F might be effective directly against certain strains of viruses. Others, like Fangchinoline, may operate via indirect mechanisms, maybe acting as an oxygen scavenger to lower oxidative stress and shield cells from damage during viral infection i.e., COVID-19 (S6 Fig).

However, emetine’s affinity for traits like the stimulation of 5-hydroxytryptamine release (S6 Fig), a neurotransmitter that controls a number of physiological processes, may indirectly improve general health and the stress response, which may have an impact on immune function. Certain viral infections that cause neurological symptoms may benefit from its antidyskinetic properties (S6 Fig).

Although the results reported here are based on well-established computational methods which have been employed in the discovery of many drugs. It has paved the way for the creation of HIV protease inhibitors such as Saquinavir [85] kinase inhibitors for cancer treatment like Imatinib [86], and antivirals including Zanamivir for influenza and Boceprevir for hepatitis C [85]. Moreover, it is crucial to take into account any prospective advantages artificial intelligence (AI) can bring. AI is advancing in the field of computational drug design by various machine learning models and it might provide improved efficiency, accuracy, and speed [87, 88] Baricitinib is a well-known instance of the finding of a potential anti-COVID-19 medication by the application of an AI-based strategy [89].

## Conclusion

In this study, selective antiviral alkaloids were analyzed as dual inhibitors of the two important proteins, namely the main protease (6LU7) and spike glycoprotein (6LZG), of the novel corona virus. The top six interacting compounds were found to show significant interactions with key residues involved in the catalytic mechanism of 6LU7, as well as substantial hydrophobic interactions with potential residues of 6LZG. The stable interactions of these ligands with the target proteins were confirmed by the MD simulation analysis. Moreover, all leads exhibited bioactivity and complied with Lipinski’s, Veber’s, and Egan’s rules, indicating their suitability for drug development. While they did not adhere to the Ghose rule. The selected six alkaloids displayed favorable ADMET properties and antiviral potential by several biological pathways, making them promising candidates for further exploration as therapeutic agents against COVID-19. *In vitro*, *in vivo*, and clinical studies are warranted to confirm their efficacy and safety. This *in-silico* study holds significant value in advancing therapeutic drug design and discovery for combating COVID-19 through naturally occurring alkaloids.

## Supporting information

S1 Fig Schematic representations and 3D visualizations of molecular interactions of standards with their respective proteins. (A) Schematic depiction of interactions for N3 with 6LU7, (B) 3D representation of N3 (blue) interactions with 6LU7 (magenta) (C) Schematic depiction of interactions for molnupiravir with 6LZG and (D) 3D representation of molnupiravir (red) interactions with 6LZG (yellow) with the representation of hydrophobic (grey circles), hydrophilic interactions (orange circles) and hydrogen bonds (yellow dashed lines).
(TIF)

S2 Fig Graphical representation of COVID-19 main protease protein (6LU7) in complex with ligands Acrimarine-f, Fangchinline and Liensinine at 0 ns and 50 ns of MD simulation. (TIF)

S3 Fig Graphical representation of Coronavirus spike receptor-binding domain (6LZG) in complex with ligands Acrimarine-f, Fangchinline and Liensinine at 0 ns and 50 ns of MD simulation. (TIF)

S4 Fig Column chart representing the predicted biological activities of Liensinine, Isoliensinine and Neferine. Each column corresponds to a specific biological activity, with the height of the colomn indicating its probability.
(TIF)

S5 Fig Column chart representing the predicted biological activities of Fangchinoline. Each column corresponds to a specific biological activity, with the height of the column indicating its probability.
(TIF)

S6 Fig Column chart representing the predicted biological activities of Acramine F (A) and Emetine (B). Each column corresponds to a specific biological activity, with the height of the colomn indicating its probability.
(TIF)

S1 Table Library of potential antiviral alkaloids screened against SARS-CoV-2 main protease and spike glycoprotein. (DOCX)

S2 Table Crystallographic properties of selected proteins (6LU7 and 6LZG). (DOCX)

S3 Table Bioactivity score of the six dual-active alkaloids evaluated by Molinspiration cheminformatics tool. (DOCX)
