# Supplementary figures and images for: In-silico evaluation of natural alkaloids against the main protease and spike glycoprotein as potential therapeutic agents for SARS-CoV-2
Source: PLoS One. 2024 Jan 4;19(1):e0294769. doi: 10.1371/journal.pone.0294769 (PMC10766191; doi:10.1371/journal.pone.0294769)

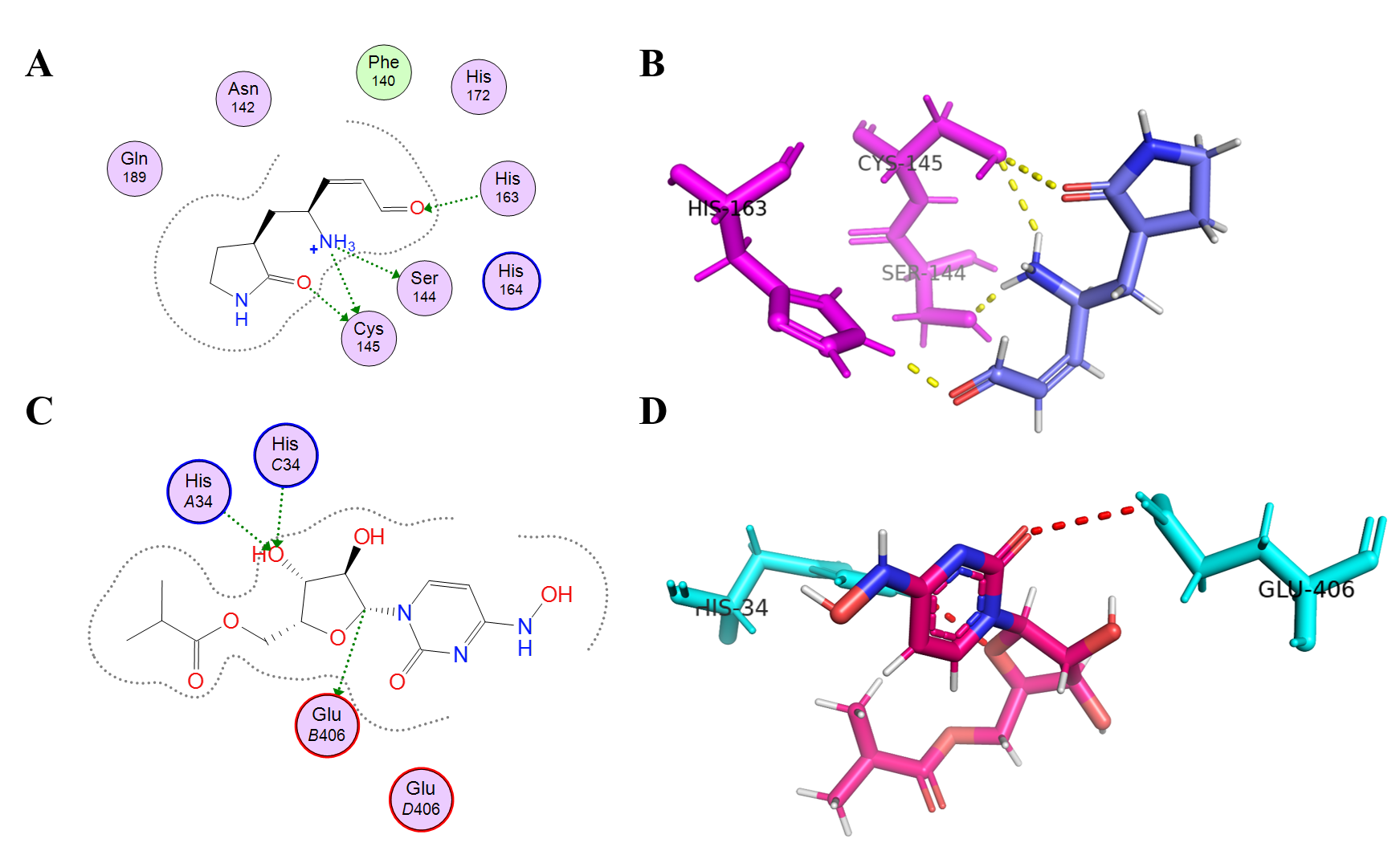

Supplement: S1 Fig — (A) Schematic depiction of interactions for N3 with 6LU7, (B) 3D representation of N3 (blue) interactions with 6LU7 (magenta) (C) Schematic depiction of interactions for molnupiravir with 6LZG and (D) 3D representation of molnupiravir (red) interactions with 6LZG (yellow) with the representation of hydrophobic (grey circles), hydrophilic interactions (orange circles) and hydrogen bonds (yellow dashed lines). (TIF) [file pone.0294769.s001.tif]

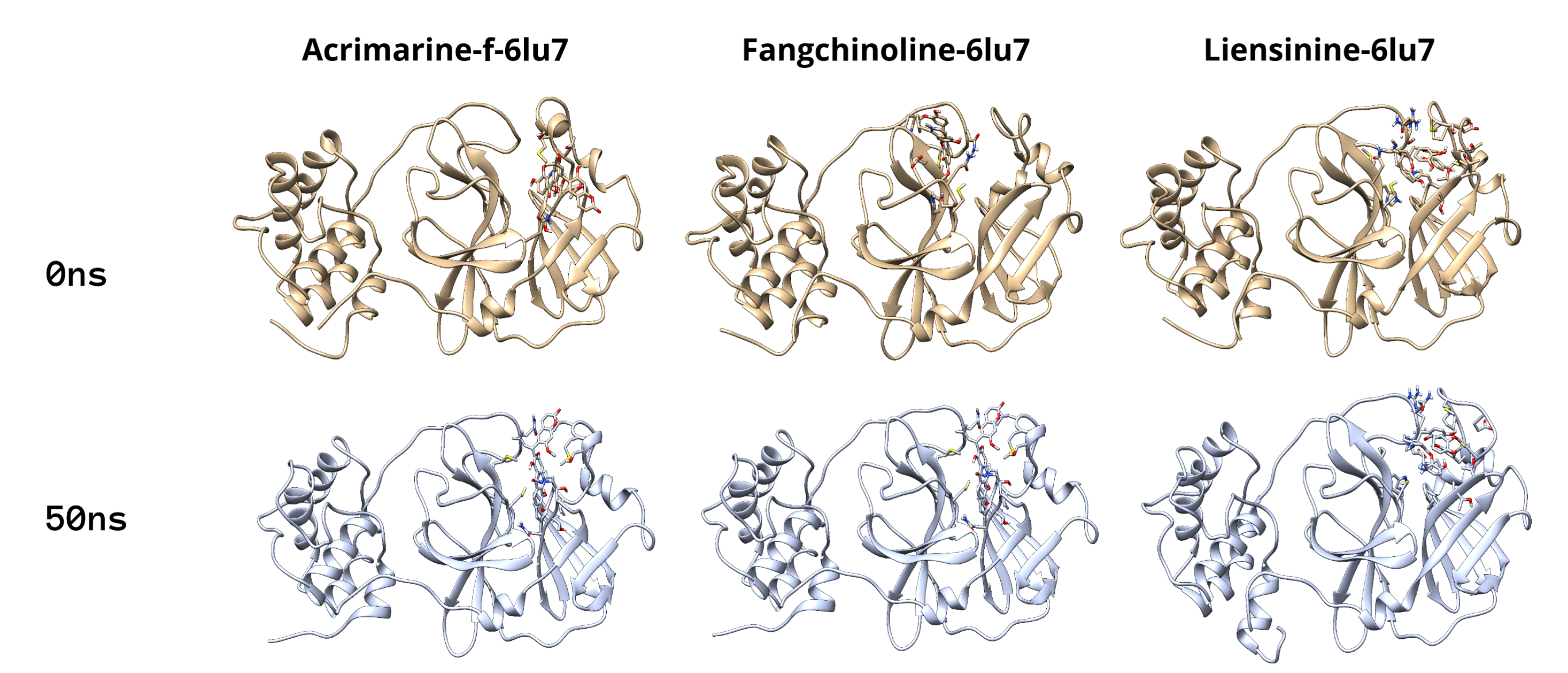

Supplement: S2 Fig — (TIF) [file pone.0294769.s002.tif]

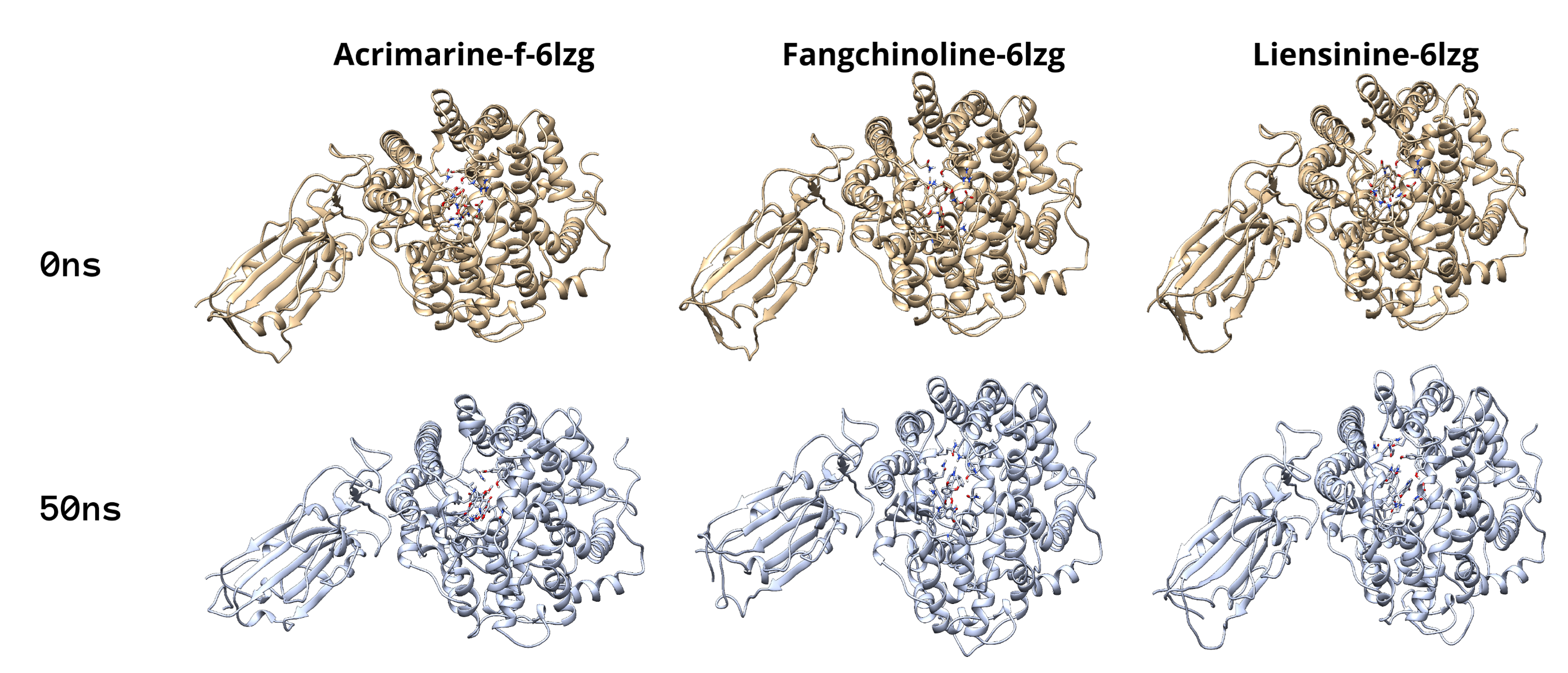

Supplement: S3 Fig — (TIF) [file pone.0294769.s003.tif]

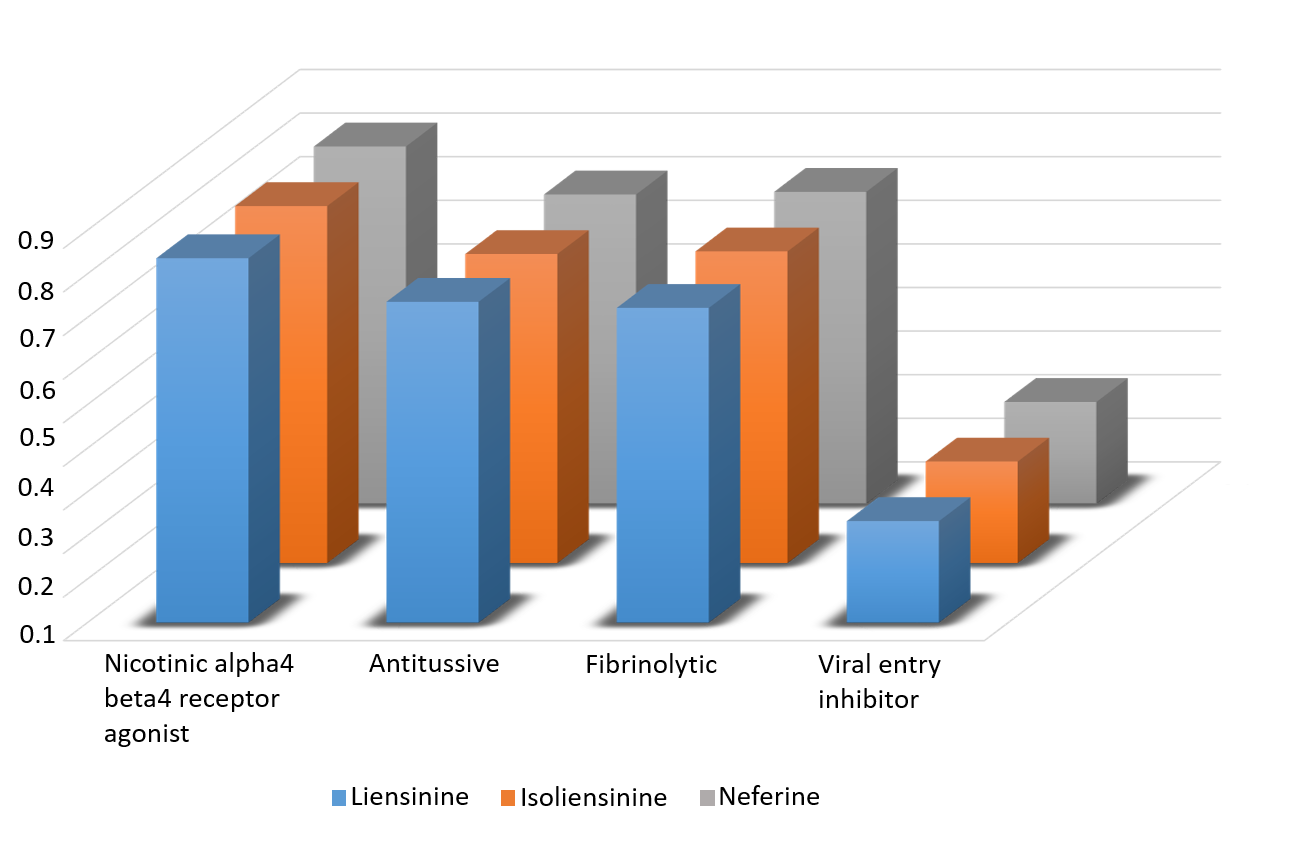

Supplement: S4 Fig — Each column corresponds to a specific biological activity, with the height of the colomn indicating its probability. (TIF) [file pone.0294769.s004.tif]

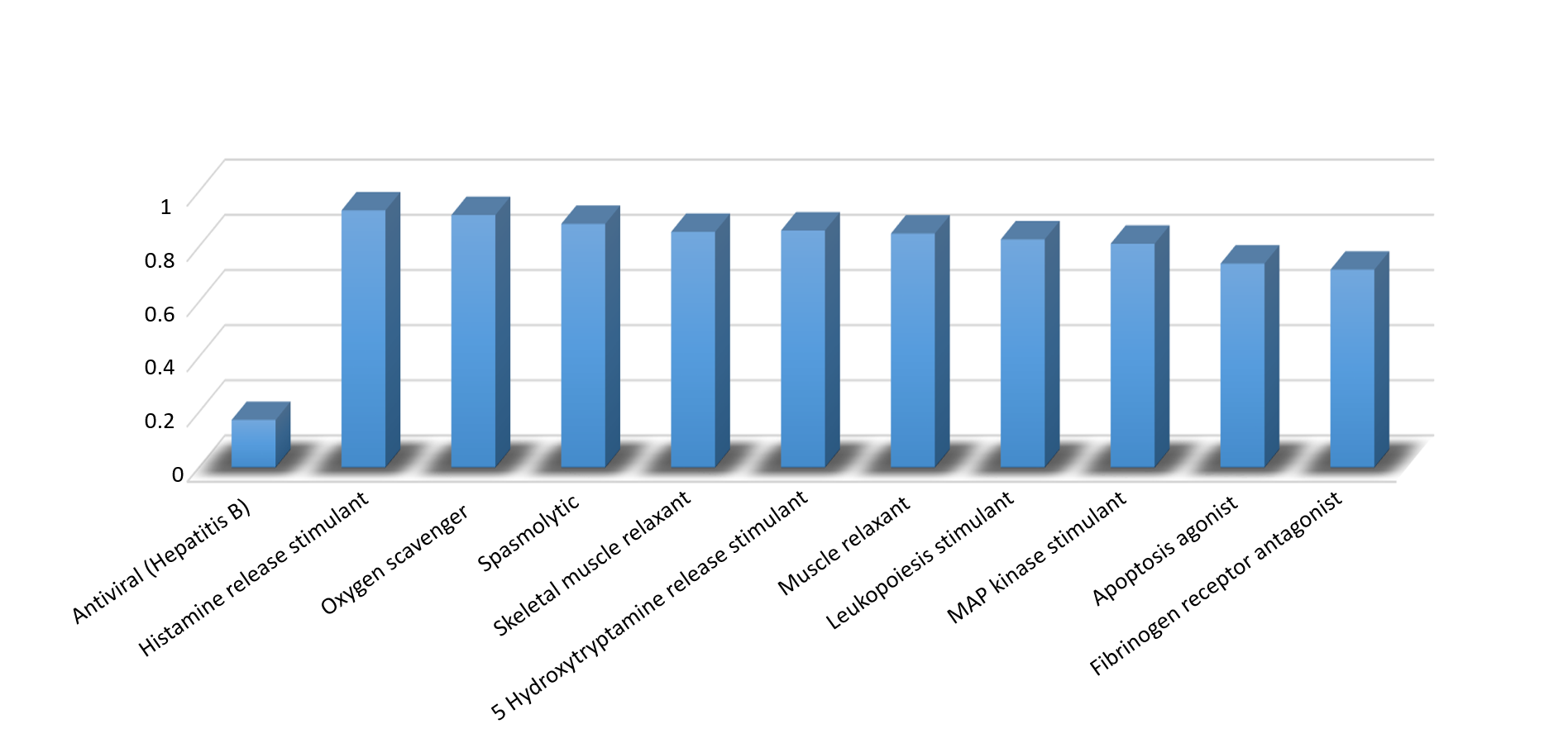

Supplement: S5 Fig — Each column corresponds to a specific biological activity, with the height of the column indicating its probability. (TIF) [file pone.0294769.s005.tif]

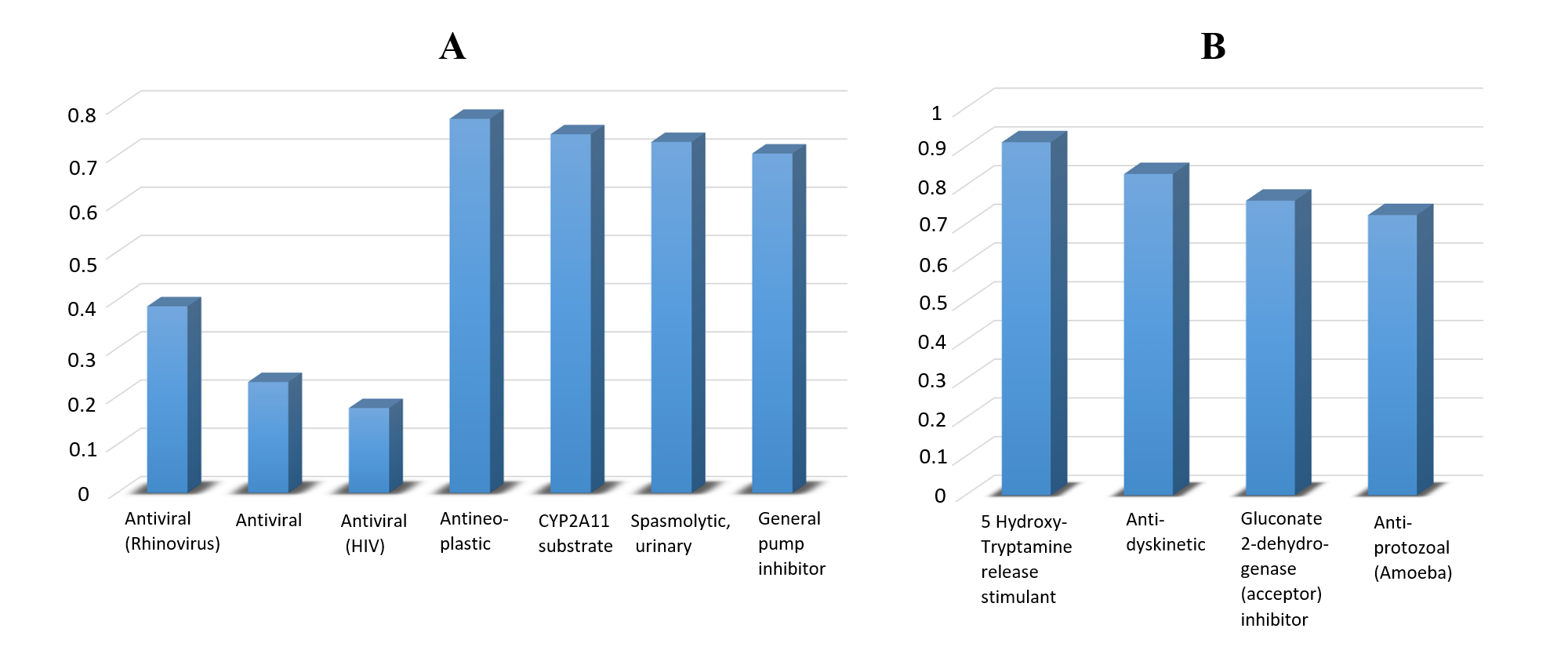

Supplement: S6 Fig — Column chart representing the predicted biological activities of Acramine F (A) and Emetine (B). Each column corresponds to a specific biological activity, with the height of the colomn indicating its probability. (TIF) [file pone.0294769.s006.tif]
