# Supplementary material for: In-silico evaluation of natural alkaloids against the main protease and spike glycoprotein as potential therapeutic agents for SARS-CoV-2
Source: PLoS One. 2024 Jan 4;19(1):e0294769. doi: 10.1371/journal.pone.0294769 (PMC10766191; doi:10.1371/journal.pone.0294769)
Supplement: S1 Table — (DOCX) [file pone.0294769.s007.docx]

**S1 Table.** Library of potential antiviral alkaloids screened against SARS-CoV-2 main protease and spike glycoprotein.

| **Sr. No.** | **Compound Structure** | **Docking Score**  **for 6lu7**  **(kcal/mol)** | **Docking Score**  **for 6lzg**  **(kcal/mol)** | **Reference** |
| --- | --- | --- | --- | --- |
| 1. |   (S)-Norcoclaurine | -5.4038 | -5.5300 | [1]  [2] |
| 2. |   (R)-Coclaurine | -4.8611 | -6.3476 | [1]  [2] |
| 3. |   (R)-N-Methylcoclaurine | -5.8657 | -6.3069 | [1]  [2] |
| 4. |   Armepavine | -5.5234 | -6.2157 | [1]  [2] |
| 5. |   Lotusine | -5.4715 | -6.3987 | [1]  [2] |
| 6. |   Reticuline | -5.5481 | -6.8320 | [1]  [3] |
| 7. |   Norjuziphine | -5.2494 | -6.3488 | [1]  [3] |
| 8. |   Liensinine | -6.5842 | -8.7930 | [1]  [2] |
| 9. |   Neferine | -7.5025 | -10.0245 | [1]  [2] |
| 10 |   Isoliensinin | -7.7072 | -9.6900 | [1]  [2] |
| 11. |   Fangchinoline | -6.2187 | -7.9562 | [1]  [4] |
| 12. | Aromoline | -5.9100 | -7.9392 | [1]  [5] |
| `13. | `  Cycleanine | -6.2874 | -7.5673 | [1]  [6] |
| 14. |   3,4-Dehydrocycleanine | -5.9387 | -7.6115 | [1]  [3] |
| 15. |   Norcycleanine | -6.2374 | -7.1152 | [1]  [3] |
| 16. |   Obaberine | -7.7053 | -7.5773 | [1]  [3] |
| 17. |   Homoaromoline | -6.4879` | -7.6599 | [1]  [3] |
| 18. |   Isotetradrine | -6.1529 | -7.7510 | [1]  [3]  [7] |
| 19. |   Berbamine | -5.3700 | -7.8207 | [1]  [3] |
| 20. |   Thalrugosine | -6.5675 | -7.4478 | [1]  [3] |
| 21. |   (+)-Stephibaberine | -5.9816 | -8.2893 | [1]  [3] |
| 22. |   Nornuciferine | -4.9456 | -6.1658 | [1]  [2] |
| 23. |   Isoboldine | -5.2212 | -6.7362 | [1]  [3]  [8]  [9] |
| 24. |   Glaucine | -6.1470 | -7.3881 | [1]  [8] |
| 25. |   N-Methyllaurotetanine | -5.7575 | -7.0941 | [1]  [8] |
| 26. |   Nuciferine | -5.4594 | -6.5771 | [1]  [8] |
| 27. |   Berberine | -5.4254 | -5.8434 | [1]  [10]  [11]  [9]  [12] |
| 28. |   Columbamine | -5.3986 | -6.3148 | [1]  [11]  [9]  [13]  [12] |
| 29. |   Jatrorrhizine | -4.9836 | -6.1183 | [1]  [10]  [11] |
| 30. |   Dehydrocavidine | -6.0030 | -6.3874 | [1]  [14] |
| 31. |   Dehydroapocavidine | -5.1576 | -6.1565 | [1]  [14] |
| 32. |   Protopine | -4.6507 | -6.0546 | [1]  [15] |
| 33. |   Allocryptopine | -5.4741 | -6.9373 | [1]  [15] |
| 34. |   Sanguinarine | -4.7642 | -6.0837 | [1]  [16] |
| 35. |   fagaronine chloride | -5.2563 | -6.6784 | [1]  [17] |
| 36. |   Nitidine chloride | -5.0336 | -6.3385 | [1]  [17] |
| 37. |   6-Acetonyldihydrochelerythrine | -6.0193 | -7.3105 | [1]  [18] |
| 38. |   Dihydrochelerythrine | -5.1995 | -6.1784 | [1]  [18] |
| 39. |   Fumarophycine | -5.2962 | -7.0006 | [1]  [19] |
| 40. |   Sibiricine | -5.9316 | -6.1961 | [1]  [19]  (Aljofan et al., 2014 |
| 41. |   Corpaine | -5.9981 | -6.9186 | [1]  [19] |
| 42. |   Dihydrofumariline | -6.0391 | -5.9773 | [1]  [19] |
| 43. |   parfumine | -5.5977 | -6.2730 | [1] |
| 44. |   Flavinantine | -4.9882 | -5.8478 | [1] |
| 45. |   Emetine | -5.9981 | -6.9185 | [1]  [20]  [9]  [12] |
| 46. |   Yohimbine | -5.1065 | -5.9315 | [21] |
| 47. |   Vincamine | -5.2636 | -6.2879 | [21] |
| 48. |   Scopolamine | -4.8301 | -5.8106 | [21]  [12] |
| 49. |   Atropine | -5.4710 | -6.3280 | [21]  [9]  [12] |
| 50. |   Colchicine | -5.7690 | -6.9115 | [21] |
| 51. |   Allantoin | -4.1139 | -3.9349 | [21] |
| 52. |   Trigonelline | -3.8905 | -3.9771 | [21] |
| 53. |   Octopamine | -4.3510 | -4.1569 | [21] |
| 54. |   Synephrine | -4.8429 | -4.8900 | [21] |
| 55. |   Capsaicin | -5.9568 | -6.7015 | [21] |
| 56. |   Citrusinine I | -5.2316 | -5.8374 | [12] |
| 57. |   Atalaphillidine | -4.9593 | -6.0683 | [12] |
| 58. |   Lycorine | -5.0160 | -5.4671 | [12]  [9] |
| 59. |   Pretazettine | -4.5903 | -5.8156 | [12]  [9] |
| 60. |   Oliverine | -5.3382 | -6.7945 | [12]  [9] |
| 61. |   Pachystaudine | -5.4311 | -6.7916 | [12]  [9] |
| 62. |   Oxostephanine | -5.2096 | -5.6639 | [9]  [12] |
| 63. |   Chelidonine | -5.1498 | -5.6652 | [9]  [12] |
| 64. |   Fagaronine | -5.9045 | -6.2049 | [9]  [12] |
| 65. |   6-Canthinone | -4.5349 | -4.9499 | [12] |
| 66. |   Harmine | -4.5627 | -4.9206 | [9]  [12] |
| 67. |   Harman | -4.3550 | -4.7536 | [12] |
| 68. |   Harmol | -4.3284 | -4.8001 | [12] |
| 69. |   O-Demethyl-buchenavianine | -5.3962 | -6.8148 | [9]  [12] |
| 70. |   Camptothecin | -4.8017 | -6.2299 | [12] |
| 71. |   Castanospermine | -3.9788 | -4.3684 | [13]  [9]  [12] |
| 72. |   Morphine | -4.1787 | -5.2554 | [12] |
| 73. |   Codeine | -4.3067 | -5.2847 | [12] |
| 74. |   Papaverine | -5.9689 | -6.7336 | [9]  [12] |
| 75. |   Cryotopleurine | -6.1998 | -6.9453 | [12] |
| 76. |   1-Deoxynojirimycin | -4.2129 | -4.0353 | [12] |
| 77. |   1-Deoxymannojirimycin | -4.0916 | -3.9257 | [12] |
| 78. |   Alpha-Homonojirimycin | -4.8784 | -4.8246 | [12] |
| 79. |   Australine | -4.3922 | -4.6368 | [12]  [13]  [9] |
| 80. |   Psychotrine | -5.9789 | -8.0166 | [9]  [12] |
| 81. |   Buchapine | -4.6728 | -6.8979 | [9]  [12] |
| 82. |   Periformyline | -5.2258 | -6.1628 | [13]  [9] |
| 83. |   Leurocristine | -5.7144 | 0 | [9]  [13] |
| 84. |   Hirsutine | -5.4310 | -6.9580 | [22]  [23] |
| 85. |   Hippeastrine | -5.6672 | -5.7862 | [22]  [24] |
| 86. |   Hemanthamine | -4.8869 | -6.3095 | [22]  [24] |
| 87. |   Matrine | -4.6680 | -5.5578 | [22]  [25] |
| 88. |   Sophocarpine | 0 | 0 | [22]  [26] |
| 89. |   Sophoridine | 0 | 0 | [22] |
| 90. |   Neoechinulin B | -5.1786 | -6.4611 | [22]  [27] |
| 91. |   Acrimarine F | -5.4310 | -6.9579 | [13]  [9] |
| 92. |   Lycoricidine | -4.8320 | -5.5101 | [9]  [28] |
| 93. |   Caribine | -5.7867 | -6.2489 | [9] |
| 94. |   Caffeine | -4.2556 | -4.3518 | [9] |
| 95. |   Harmaline | -4.8254 | -5.0778 | [9] |
| 96. |   Buxamine E | -4.8623 | 0 | [9] |
| 97. |   Narciclasine | -4.6630 | -5.7369 | [9] |
| 98. | N-Methyllaurotetanine | -5.1811 | -7.0490 | [9] |
| 99. |   10-Methoxycamptothecin | -6.0662 | -6.9097 | [9] |
| 100. |   Odorinol | -5.5792 | -6.5125 | [9] |
| 101. | Taspine | -5.9241 | -6.6662 | [9] |
| 102. | Pancratistatin | -5.0087 | -5.7358 | [9] |

**References**

1. Qing Z-X, Yang P, Tang Q, Cheng P, Liu X-B, Zheng Y, et al. Isoquinoline Alkaloids and Their Antiviral, Antibacterial, and Antifungal Activities and Structure-activity Relationship. Current Organic Chemistry. 2017;21. doi:10.2174/1385272821666170207114214

2. Kashiwada Y, Aoshima A, Ikeshiro Y, Chen YP, Furukawa H, Itoigawa M, et al. Anti-HIV benzylisoquinoline alkaloids and flavonoids from the leaves of Nelumbo nucifera, and structure-activity correlations with related alkaloids. Bioorganic and Medicinal Chemistry. 2005;13: 443–448. doi:10.1016/J.BMC.2004.10.020

3. Nawawi A, Ma CM, Nakamura N, Hattori M, Kurokawa M, Shiraki K, et al. Anti-herpes simplex virus activity of alkaloids isolated from Stephania cepharantha. Biological & pharmaceutical bulletin. 1999;22: 268–274. doi:10.1248/BPB.22.268

4. Wan Z, Lu Y, Liao Q, Wu Y, Chen X. Fangchinoline inhibits human immunodeficiency virus type 1 replication by interfering with gp160 proteolytic processing. PloS one. 2012;7. doi:10.1371/JOURNAL.PONE.0039225

5. Ma CM, Nakamura N, Miyashiro H, Hattori M, Komatsu K, Kawahata T, et al. Screening of Chinese and Mongolian herbal drugs for anti-human immunodeficiency virus type 1 (HIV-1) activity. Phytotherapy research : PTR. 2002;16: 186–189. doi:10.1002/PTR.922

6. Otshudi AL, Apers S, Pieters L, Claeys M, Pannecouque C, De Clercq E, et al. Biologically active bisbenzylisoquinoline alkaloids from the root bark of Epinetrum villosum. Journal of Ethnopharmacology. 2005;102: 89–94. doi:10.1016/j.jep.2005.05.021

7. Zeng X, Dong Y, Sheng G, Dong X, Sun X, Fu J. Isolation and structure determination of anti-influenza component from Mahonia bealei. Journal of ethnopharmacology. 2006;108: 317–319. doi:10.1016/J.JEP.2006.05.014

8. Boustie J, Stigliani JL, Montanha J, Amoros M, Payard M, Girret L. Antipoliovirus structure - Activity relationships of some aporphine alkaloids. Journal of Natural Products. 1998;61: 480–484. doi:10.1021/NP970382V

9. Perez G. RM. Antiviral activity of compounds isolated from plants. Pharmaceutical Biology. 2003;41: 107–157. doi:10.1076/phbi.41.2.107.14240

10. Bodiwala HS, Sabde S, Mitra D, Bhutani KK, Singh IP. Synthesis of 9-substituted derivatives of berberine as anti-HIV agents. European Journal of Medicinal Chemistry. 2011;46: 1045–1049. doi:10.1016/j.ejmech.2011.01.016

11. Ng TB, Huang B, Fong WP, Yeung HW. Anti-human immunodeficiency virus (anti-HIV) natural products with special emphasis on HIV reverse transcriptase inhibitors. Life Sciences. 1997;61: 933–949. doi:10.1016/S0024-3205(97)00245-2

12. El Sayed KA. Natural products as antiviral agents. Studies in Natural Products Chemistry. 2000;24: 473–572. doi:10.1016/S1572-5995(00)80051-4

13. Bufo SA, Karaman R. Herbivores, Cancerous Cells and Pathogens. Toxins. 2019;11: 1–28.

14. Li HL, Han T, Liu RH, Zhang C, Chen HS, Zhang WD. Alkaloids from Corydalis saxicola and their anti-hepatitis B virus activity. Chemistry and Biodiversity. 2008;5: 777–783. doi:10.1002/CBDV.200890074

15. Aljofan M, Netter HJ, Aljarbou AN, Hadda T Ben, Orhan IE, Sener B, et al. Anti-hepatitis B activity of isoquinoline alkaloids of plant origin. Archives of Virology. 2014;159: 1119–1128. doi:10.1007/S00705-013-1937-7

16. Cheng T-J, Goodsell D, Kan C-C. Identification of Sanguinarine as a Novel HIV Protease Inhibitor from High-Throughput Screening of 2,000 Drugs and Natural Products with a Cell-Based Assay. Letters in Drug Design & Discovery. 2005;2: 364–371. doi:10.2174/1570180054405811

17. Tan GT, Miller JF, Kinghorn AD, Hughes SH, Pezzuto JM. HIV-1 and HIV-2 reverse transcriptases: A comparative study of sensitivity to inhibition by selected natural products. Biochemical and Biophysical Research Communications. 1992;185: 370–378. doi:10.1016/S0006-291X(05)80995-7

18. Tan GT, Pezzuto JM, Kinghorn AD, Hughes SH. Evaluation of natural products as inhibitors of human immunodeficiency virus type 1 (hiv-1) reverse transcriptase. Journal of Natural Products. 1991;54: 143–154. doi:10.1021/NP50073A012

19. Orhan I, Özcelik B, Şener B. Antiviral and Antimicrobial Evaluation of Some Heterocyclic Compounds from Turkish Plants. 2007; 303–323. doi:10.1007/7081_2007_072

20. Valadão ALC, Abreu CM, Dias JZ, Arantes P, Verli H, Tanuri A, et al. Natural plant alkaloid (Emetine) inhibits HIV-1 replication by interfering with reverse transcriptase activity. Molecules. 2015;20: 11474–11489. doi:10.3390/molecules200611474

21. Özçelik B, Kartal M, Orhan I. Cytotoxicity, antiviral and antimicrobial activities of alkaloids, flavonoids, and phenolic acids. Pharmaceutical Biology. 2011;49: 396–402. doi:10.3109/13880209.2010.519390

22. Moradi MT, Karimi A, Lorigooini Z. Alkaloids as the natural anti-influenza virus agents: a systematic review. Toxin Reviews. 2017;37: 11–18. doi:10.1080/15569543.2017.1323338

23. Takayama H, Iimura Y, Kitajima M, Aimi N, Konno K, Inoue H, et al. Discovery of anti-influenza A virus activity of a corynanthe-type indole alkaloid, hirsutine, in vitro and the structure-activity relationship of natural and synthetic analogs. Bioorganic and Medicinal Chemistry Letters. 1997;7: 3145–3148. doi:10.1016/S0960-894X(97)10154-8

24. He J, Qi WB, Wang L, Tian J, Jiao PR, Liu GQ, et al. Amaryllidaceae alkaloids inhibit nuclear-to-cytoplasmic export of ribonucleoprotein (RNP) complex of highly pathogenic avian influenza virus H5N1. Influenza and Other Respiratory Viruses. 2013;7: 922–931. doi:10.1111/IRV.12035

25. Dang Z, Jung K, Zhu L, Lai W, Xie H, Lee KH, et al. Identification and synthesis of quinolizidines with anti-influenza A virus activity. ACS Medicinal Chemistry Letters. 2014;5: 942–946. doi:10.1021/ML500236N

26. Pan QM, Li YH, Hua J, Huang FP, Wang HS, Liang D. Antiviral Matrine-Type Alkaloids from the Rhizomes of Sophora tonkinensis. Journal of Natural Products. 2015;78: 1683–1688. doi:10.1021/ACS.JNATPROD.5B00325

27. Chen X, Si L, Liu D, Proksch P, Zhang L, Zhou D, et al. Neoechinulin B and its analogues as potential entry inhibitors of influenza viruses, targeting viral hemagglutinin. European Journal of Medicinal Chemistry. 2015;93: 182–195. doi:10.1016/j.ejmech.2015.02.006

28. Song B, Yang S, Jin L, Bhadury P. Environment-friendly antiviral agents for plants. 2011.
