# Supplementary material for: In-silico evaluation of natural alkaloids against the main protease and spike glycoprotein as potential therapeutic agents for SARS-CoV-2
Source: PLoS One. 2024 Jan 4;19(1):e0294769. doi: 10.1371/journal.pone.0294769 (PMC10766191; doi:10.1371/journal.pone.0294769)
Supplement: S2 Table — (DOCX) [file pone.0294769.s008.docx]

**S2 Table.** Crystallographic properties of selected proteins (6LU7 and 6LZG)

| **Enzyme** | **PDB ID** | **Classification** | **Virus** | **Expression system** | **Resolution** | **Method** | **Total structural weight** | **Total chains; unique chains** |
| --- | --- | --- | --- | --- | --- | --- | --- | --- |
| Main protease | 6LU7 | Viral protein | SARS-COV2 | Escherichia coli BL21 | 2.20A | X ray diffraction | 34.5 kDa | A B C D; 2 |
| Coronavirus spike receptor binding domain in complex with ACE2 | 6LZG | Viral protein | SARS-COV2 | Insect cell expression vector pTIE1 | 2.50 A | X ray diffraction | 93.5 kDa | A B; 2 |
