# Supplementary material for: In-silico evaluation of natural alkaloids against the main protease and spike glycoprotein as potential therapeutic agents for SARS-CoV-2
Source: PLoS One. 2024 Jan 4;19(1):e0294769. doi: 10.1371/journal.pone.0294769 (PMC10766191; doi:10.1371/journal.pone.0294769)
Supplement: S3 Table — (DOCX) [file pone.0294769.s009.docx]

**S3 Table.** Bioactivity score of the six dual-active alkaloids evaluated by Molinspiration cheminformatics tool.

| **Sr. No.** | **Compound name** | **GPCR ligand** | **Ion channel modulator** | **Kinase inhibitor** | **Nuclear receptor ligand** | **Protease inhibitor** | **Enzyme inhibitor** |
| --- | --- | --- | --- | --- | --- | --- | --- |
| 1 | Liensinine | -0.01 | -0.62 | -0.43 | -0.46 | -0.04 | -0.34 |
| 2 | Neferine | -0.11 | -0.76 | -0.55 | -0.59 | -0.09 | -0.45 |
| 3 | Isoliensinine | -0.03 | -0.63 | -0.45 | -0.48 | -0.05 | -0.35 |
| 4 | Fangchinoline | -0.02 | -0.61 | -0.49 | -0.54 | -0.07 | -0.30 |
| 5 | Emetine | 0.22 | 0.10 | -0.27 | -0.20 | 0.08 | -0.06 |
| 6 | Acrimarine F | -0.11 | -0.41 | -0.28 | 0.01 | -0.36 | 0.15 |
